# Supplementary material for: Hydrogen Delocalization in an Asymmetric Biomolecule: The Curious Case of Alpha-Fenchol
Source: Molecules. 2021 Dec 24;27(1):101. doi: 10.3390/molecules27010101 (PMC8746872; doi:10.3390/molecules27010101)
Supplement: Supplementary file 1 [file molecules-27-00101-s001.zip › molecules-1480529-supplementary.pdf]

Hydrogen Delocalization in an Asymmetric Biomolecule: The Curious Case of alpha-Fenchol
-
Electronic Supplementary Information

Robert Medel[1,*], Johann R. Springborn[1], Deborah L. Crittenden[2] and Martin A. Suhm[1]

Contents

1 Calculations and Spectra for Borneol 3

2 Calculations and Spectra for Isopinocampheol 7

3 Localized Conformers of α- and β-Fenchol 11
3.1 OH Stretch Spectroscopic Properties of α- and β-Fenchol 11
3.2 Comparison of a Simulation for a Mixture of α- and β-Fenchol with Experiment . 12

4 Experimental OH Stretch Fundamental Wavenumbers for Alcohol Conformers and Dimer Acceptors Used in the Training Set for the Model for the Isotope Effect 13

5 Estimated Bond Length Extension Factors 14

6 Validation of the 1D Torsion Code for Symmetric Alcohols 15
6.1 Overview 15
6.2 Methanol 16
6.2.1 Methanol CH_3OH/D 16
6.2.2 Methanol CD_3OH/D 17
6.3 Primary Alcohols 18
6.3.1 Ethanol 18
6.3.2 Propargyl Alcohol 19
6.3.3 2-Hydroxyacetonitrile 20
6.3.4 4-Hydroxy-2-butynenitrile 21
6.3.5 Allyl Alcohol 22
6.3.6 Methoxymethanol 23
6.3.7 Fluoromethanol 24
6.3.8 2,2,2-Trifluoroethanol 25
6.4 Secondary Alcohols 26
6.4.1 2-Propanol 26
6.4.2 Equatorial Cyclohexanol 27
6.4.3 Cyclopropanol 28
6.5 Tertiary Alcohols 29
6.5.1 *tert*-Butyl Alcohol 29
6.5.2 Axial 1-Methylcyclohexanol 30

[1]Institute of Physical Chemistry, University of Goettingen, Tammannstr. 6, 37077 Goettingen. Germany. E-mail: rmedel@gwdg.de, johann-richard.springborn@chemie.uni-goettingen.de, msuhm@gwdg.de

[2]School of Physical and Chemical Sciences, University of Canterbury, Private Bag 4800, Christchurch 8140, New Zealand E-mail: deborah.crittenden@canterbury.ac.nz

6.5.3 Equatorial 1-Methylcyclohexanol . . . 31
6.5.4 1-Vinylcyclopropanol . . . 32

7 Torsional Modelling of (+)-α-Fenchol 33
7.1 Calculated Properties of the Stationary Points of the Torsional Potential for (+)-α-Fenchol . . . 33
7.2 Torsional Potentials for (+)-α-Fenchol . . . 34
7.2.1 Ground State at B3LYP-D3(BJ) Level . . . 34
7.2.2 Symmetrized Ground State at B3LYP-D3(BJ) Level . . . 35
7.2.3 Symmetrized Ground State at DLPNO-CCSD(T)//B3LYP-D3(BJ) Level . 36
7.2.4 Ground State at Zero-point Corrected DLPNO-CCSD(T)//B3LYP-D3(BJ) Level . . . 37
7.2.5 OH/OD Stretch Excited State at DLPNO-CCSD(T)/aug-cc-pVQZ//B3LYP-D3(BJ) Level and Franck-Condon Factors . . . 38
7.2.6 OH/OD Stretch Excited State at Zero-point Corrected DLPNO-CCSD(T)/aug-cc-pVQZ//B3LYP-D3(BJ) Level and Franck-Condon Factors . . . 40
7.2.7 Artificial Localization of the Ground State at DLPNO-CCSD(T)/aug-cc-pVQZ// B3LYP-D3(BJ) Level . . . 42
7.2.8 Artificial Localization of the OH/OD Stretch Excited State at DLPNO-CCSD(T)/ aug-cc-pVQZ//B3LYP-D3(BJ) Level . . . 44
7.3 Dependence of Torsional Properties on the Asymmetry . . . 45
7.3.1 (De-)Localization . . . 45
7.3.2 Franck-Condon Factors . . . 46
7.3.3 Torsional Splitting . . . 47

8 Modelling of Methanol in the OH Stretch Ground and Excited State 48

9 Modelling of Propargyl Alcohol in the OH Stretch Ground and Excited State as well as Franck-Condon Factors 49

10 List of Isomers of Propargyl Alcohol Dimers 51

11 Concentration Dependence of the FTIR Jet Spectrum of Propargyl Alcohol 52

12 Leading Isomers of (+)-α-Fenchol Dimers 53

13 Calculation of Raman Cross Sections 54

14 Used Keywords for Calculations 55
14.1 Gaussian 09 Rev. E.01 . . . 55
14.2 ORCA version 4.2.1 . . . 55

15 Experimental Band Positions and Assignments 56

1 Calculations and Spectra for Borneol

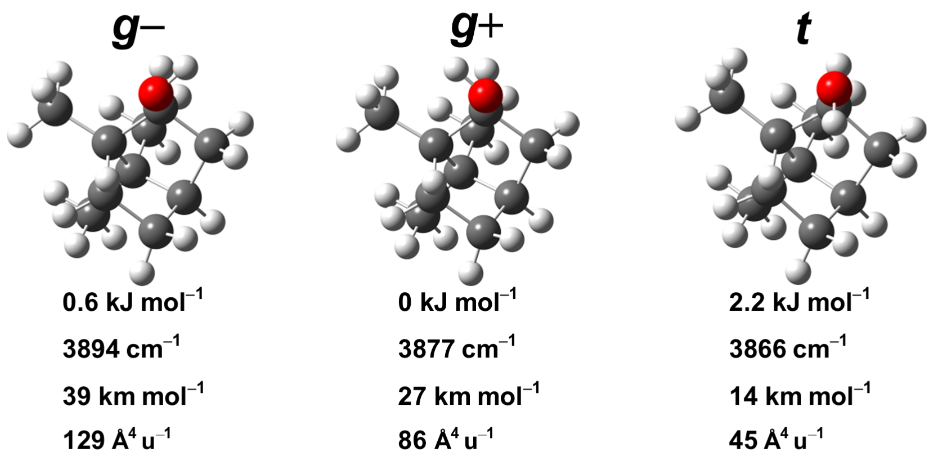

Figure S1: Structures, relative harmonically zero-point corrected energies, uncorrected harmonic OH stretching wavenumbers, IR band strengths and Raman activities for conformers of (+)-borneol at DLPNO-CCSD(T)/aug-cc-pVQZ//PBE0-D3(BJ)/may-cc-pVTZ level.

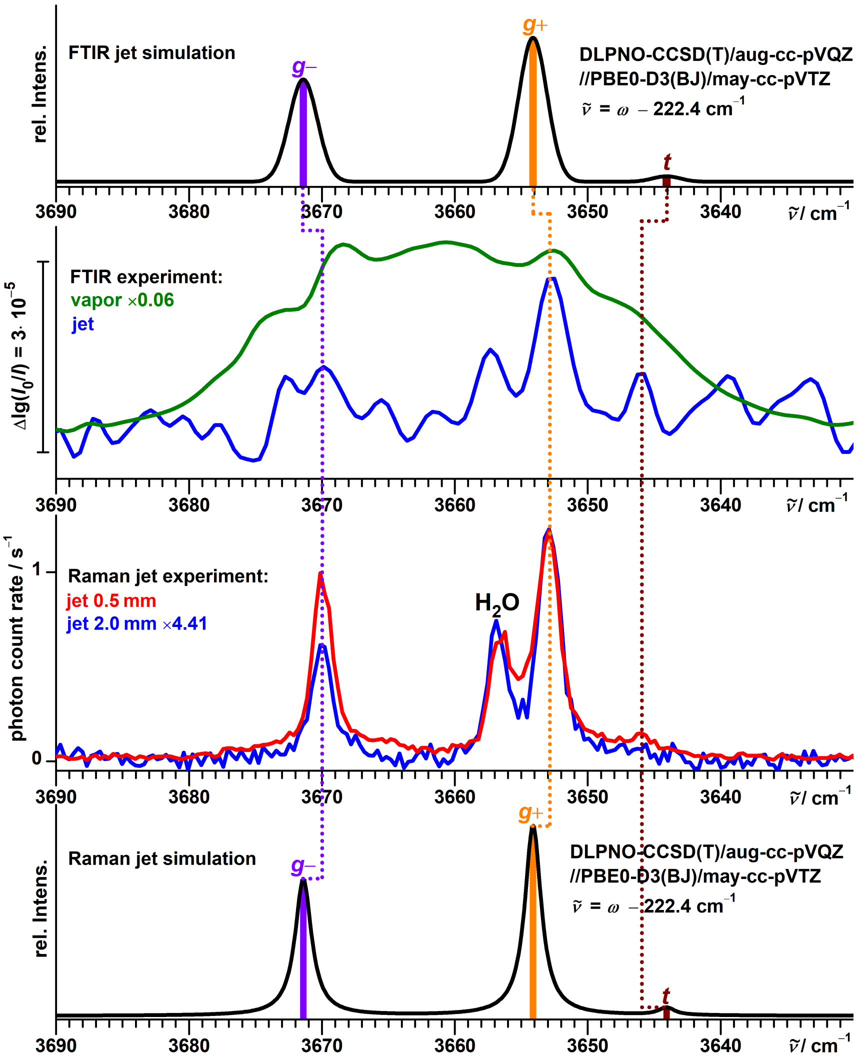

Figure S2: Top half: Comparison between simulated and experimental FTIR jet spectra of (+)-borneol. Also shown is an FTIR spectrum of the vapor at ambient temperature. Bottom half: Comparison between simulated and experimental Raman jet spectra of (+)-borneol at two different detection distances from the nozzle. The simulations assume a Boltzmann distribution of localized conformers at a conformational temperature of 100 K. Harmonic OH stretching wavenumbers are uniformly shifted according to the model based on PBE0 of Ref. 1. Note that experimentally (−)-borneol was used, but computationally (+)-borneol was analyzed for easier comparison with (+)-α-fenchol.

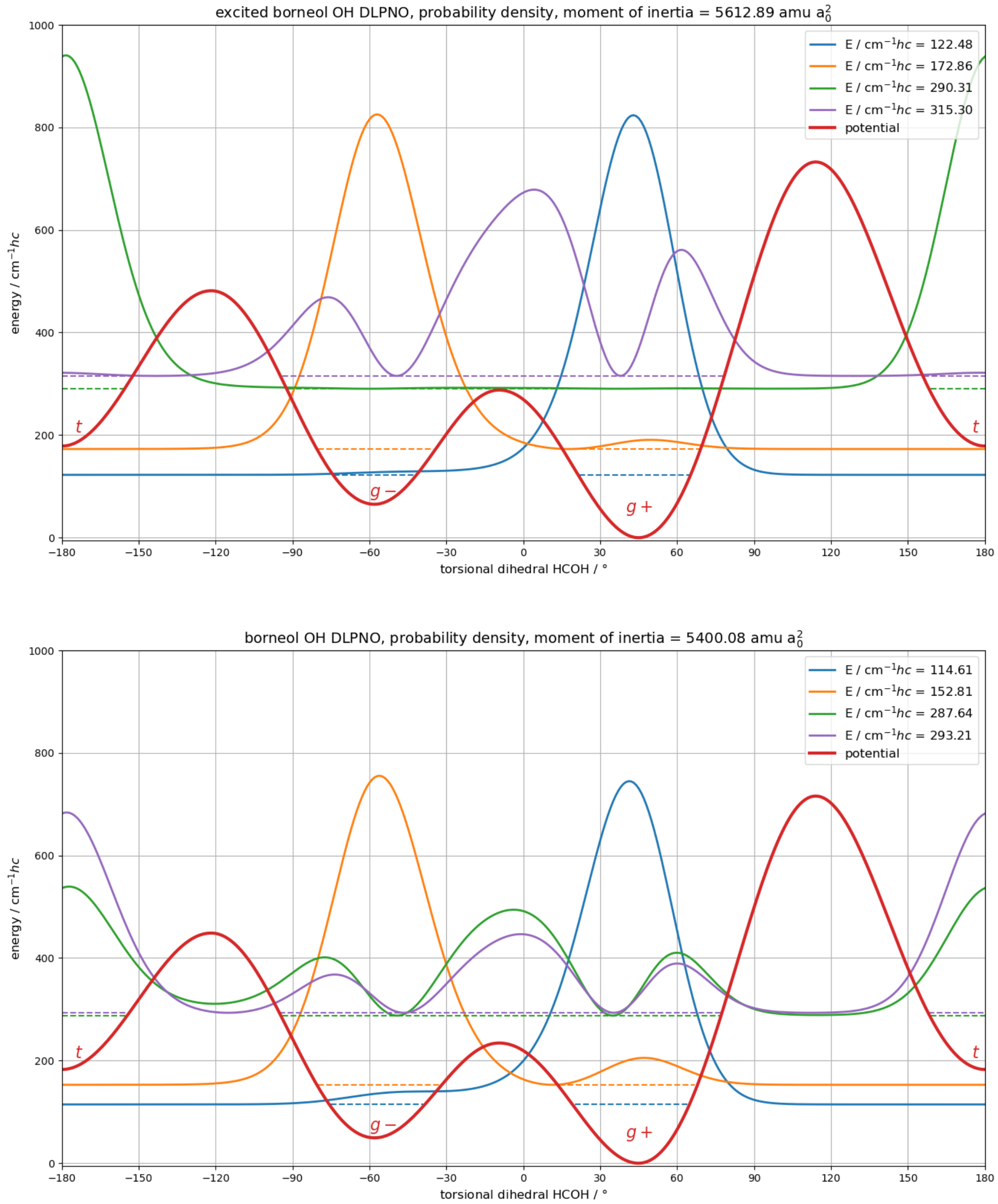

Figure S3: Bottom: Electronic torsional potential (red trace) of (+)-borneol calculated at B3LYP-D3(BJ)/may-cc-pVTZ level and scaled to DLPNO-CCSD(T)/aug-cc-pVQZ single-point corrections for the six stationary points. A constant moment of inertia, based on the $g+$ minimum geometry, was used. An accidental near-degeneracy between the third and fourth torsional state is calculated, leading to delocalization. Due to the low vapor pressure and thermal population no experimental confirmation or refutation for this feature is possible with the used setups.
Top: Estimated torsional potential for the OH stretch excited state, obtained by also adding the harmonic OH stretching wavenumbers at the stationary points before scaling. The accidental degeneracy is lost, the resonant state mixing quenched.

Table S1: Franck-Condon factors $\left\langle \chi^1_{m'} \chi^0_m \right\rangle^2$ for (+)-borneol based on Figure S3.

	χ^1_1	χ^1_2	χ^1_3
χ^0_1	0.983	$1.25 \cdot 10^{-2}$	$2.42 \cdot 10^{-5}$
χ^0_2	$1.30 \cdot 10^{-2}$	0.985	$3.65 \cdot 10^{-6}$
χ^0_3	$2.17 \cdot 10^{-3}$	$7.97 \cdot 10^{-4}$	0.455

2 Calculations and Spectra for Isopinocampheol

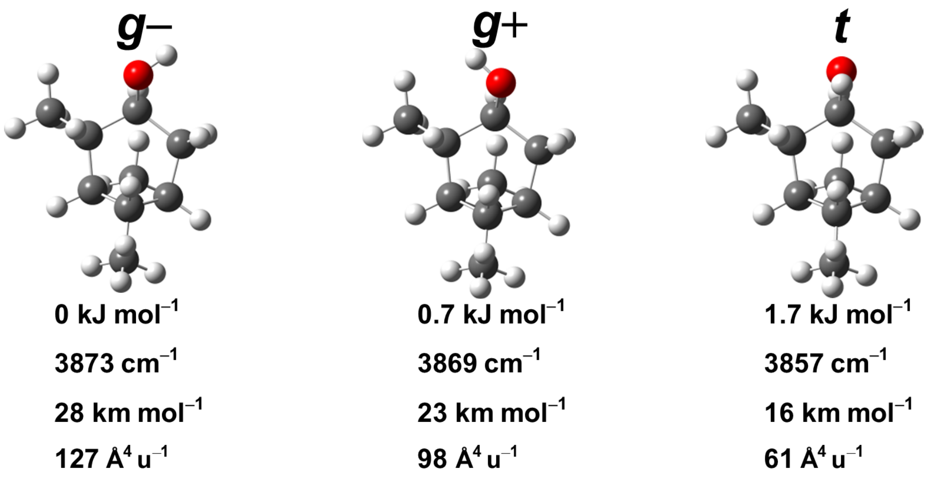

Figure S4: Structures, relative harmonically zero-point corrected energies, uncorrected harmonic OH stretching wavenumbers, IR band strengths and Raman activities for conformers of (+)-isopinocampheol at DLPNO-CCSD(T)/aug-cc-pVQZ//PBE0-D3(BJ)/may-cc-pVTZ level.

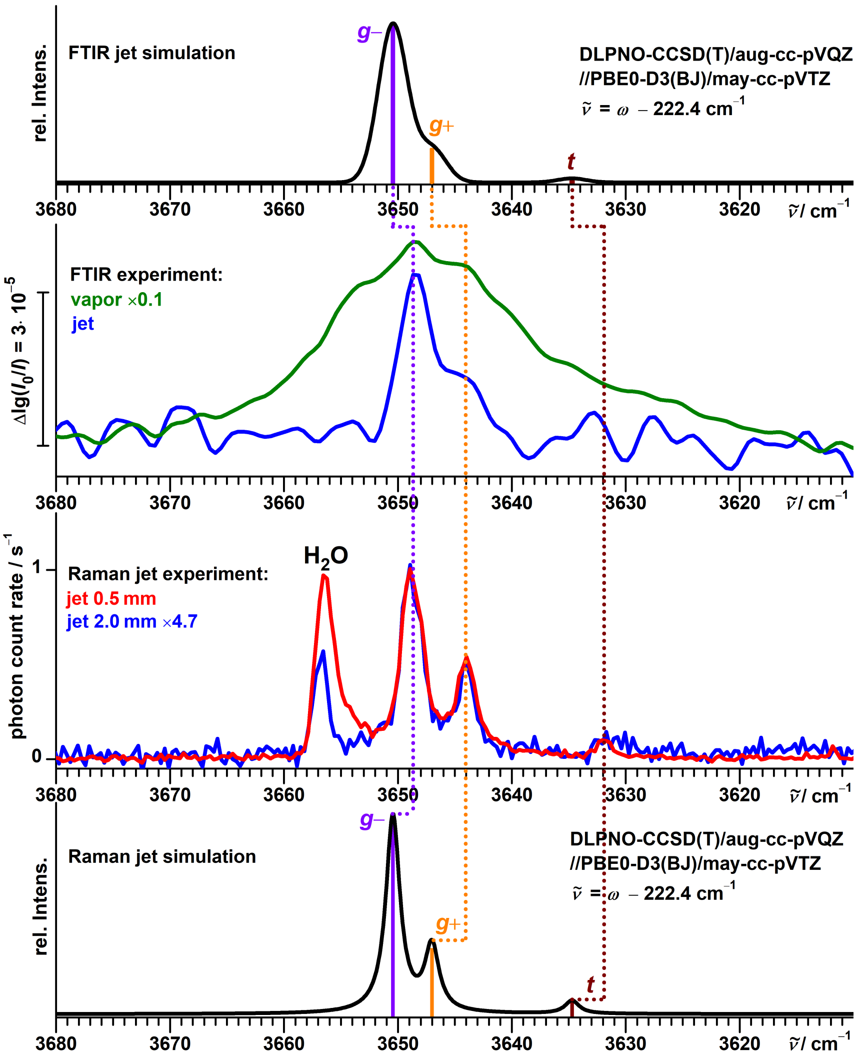

Figure S5: Top half: Comparison between simulated and experimental FTIR jet spectra of (+)-isopinocampheol. Also shown is an FTIR spectrum of the vapor at ambient temperature. Bottom half: Comparison between simulated and experimental Raman jet spectra of (+)-isopinocampheol at two different detection distances from the nozzle. The simulations assume a Boltzmann distribution of localized conformers at a conformational temperature of 100 K. Harmonic OH stretching wavenumbers are uniformly shifted according to the model based on PBE0 of Ref. 1. Note that experimentally (−)-isopinocampheol was used, but computationally (+)-isopinocampheol was analyzed for easier comparison with (+)-α-fenchol.

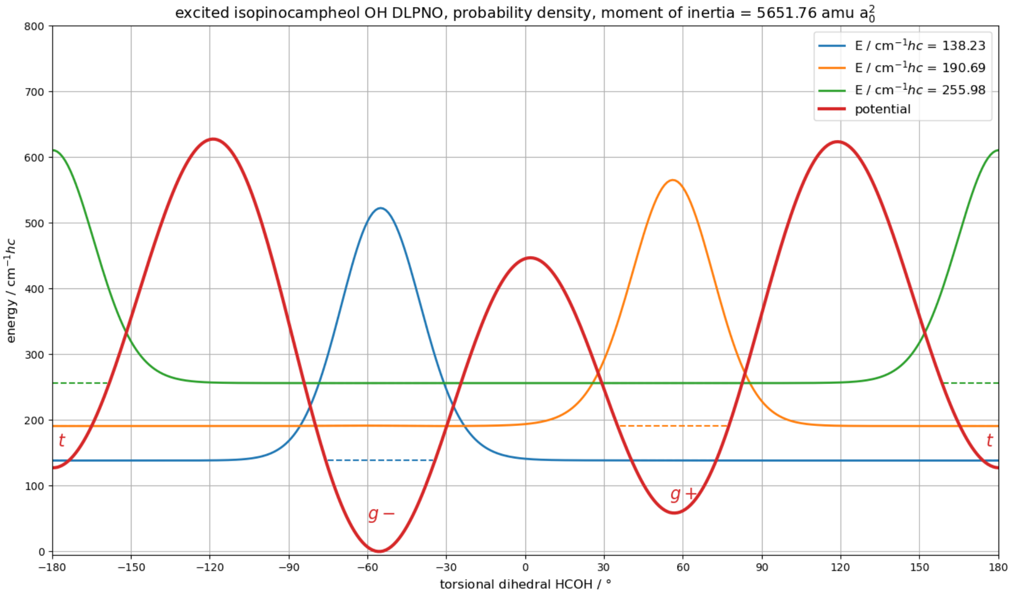

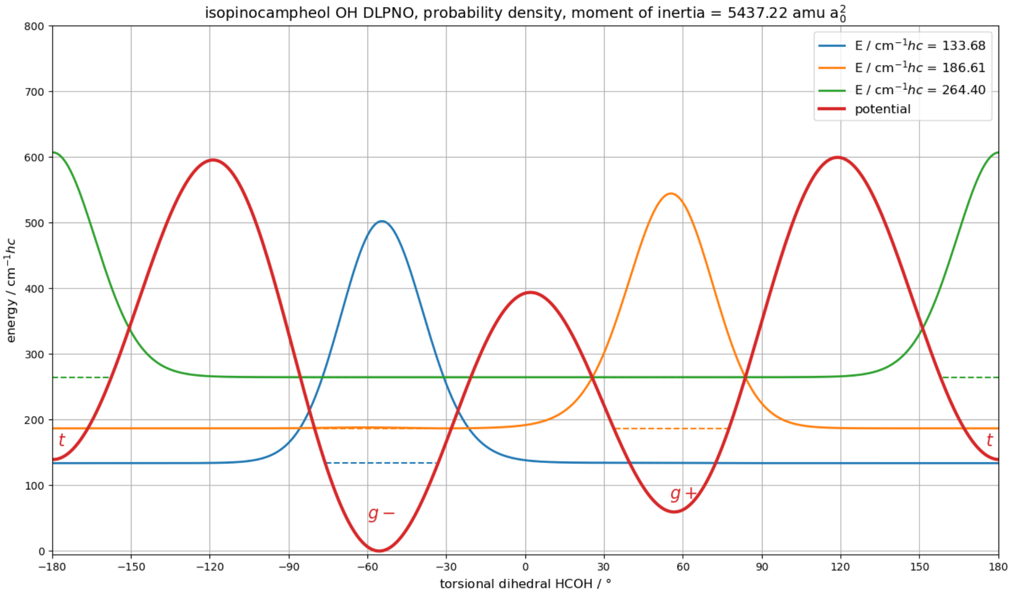

Figure S6: Bottom: Electronic torsional potential (red trace) of (+)-isopinocampheol calculated at B3LYP-D3(BJ)/may-cc-pVTZ level and scaled to DLPNO-CCSD(T)/aug-cc-pVQZ single-point corrections for the six stationary points. A constant moment of inertia, based on the $g+$ minimum geometry, was used.
Top: Estimated torsional potential for the OH stretch excited state, obtained by also adding the harmonic OH stretching wavenumbers at the stationary points before scaling.

Table S2: Franck-Condon factors $\langle \chi^1_{m'} \chi^0_m \rangle^2$ for (+)-isopinocampheol based on Figure S6.

	χ^1_1	χ^1_2	χ^1_3
χ^0_1	0.998	$2.37 \cdot 10^{-4}$	$1.38 \cdot 10^{-7}$
χ^0_2	$2.65 \cdot 10^{-4}$	0.998	$3.08 \cdot 10^{-10}$
χ^0_3	$7.16 \cdot 10^{-8}$	$3.40 \cdot 10^{-8}$	0.908

3 Localized Conformers of α- and β-Fenchol

3.1 OH Stretch Spectroscopic Properties of α- and β-Fenchol

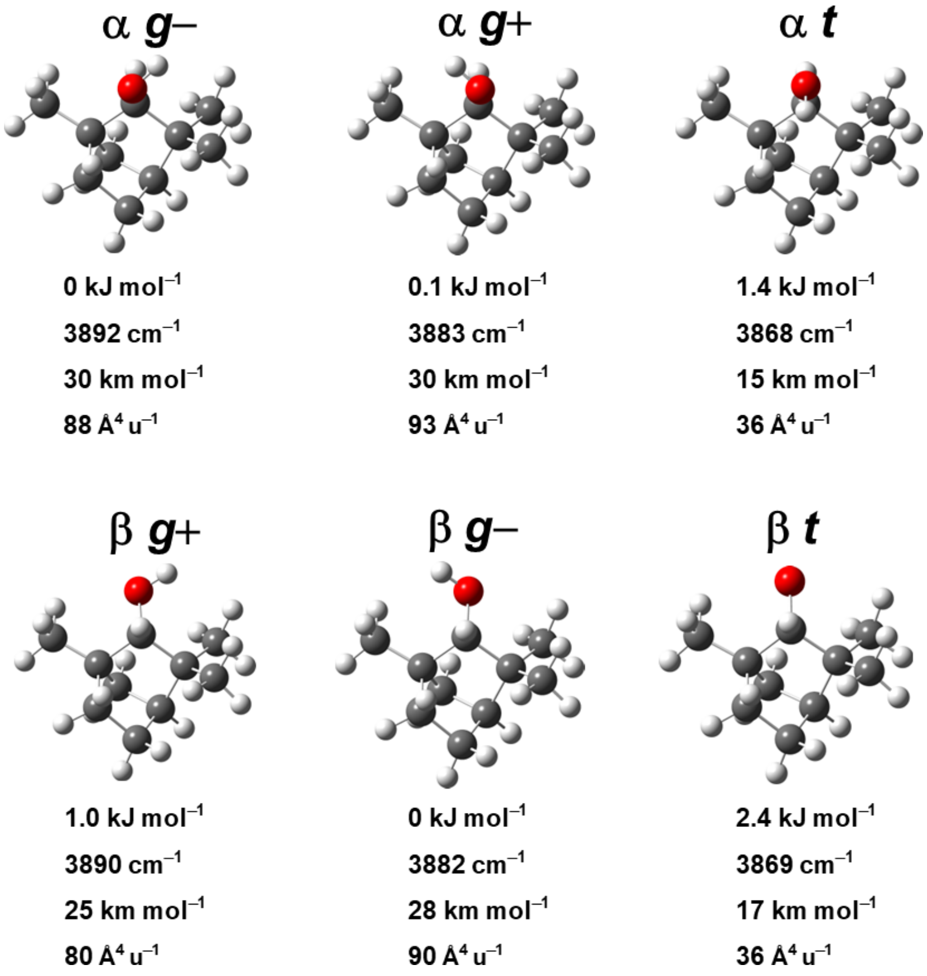

Figure S7: Structures, relative harmonically zero-point corrected energies (within each epimer), uncorrected harmonic OH stretching wavenumbers, IR band strengths and Raman activities for conformers of (+)-α- and (+)-β-fenchol at DLPNO-CCSD(T)/aug-cc-pVQZ//PBE0-D3(BJ)/may-cc-pVTZ level.

3.2 Comparison of a Simulation for a Mixture of α- and β-Fenchol with Experiment

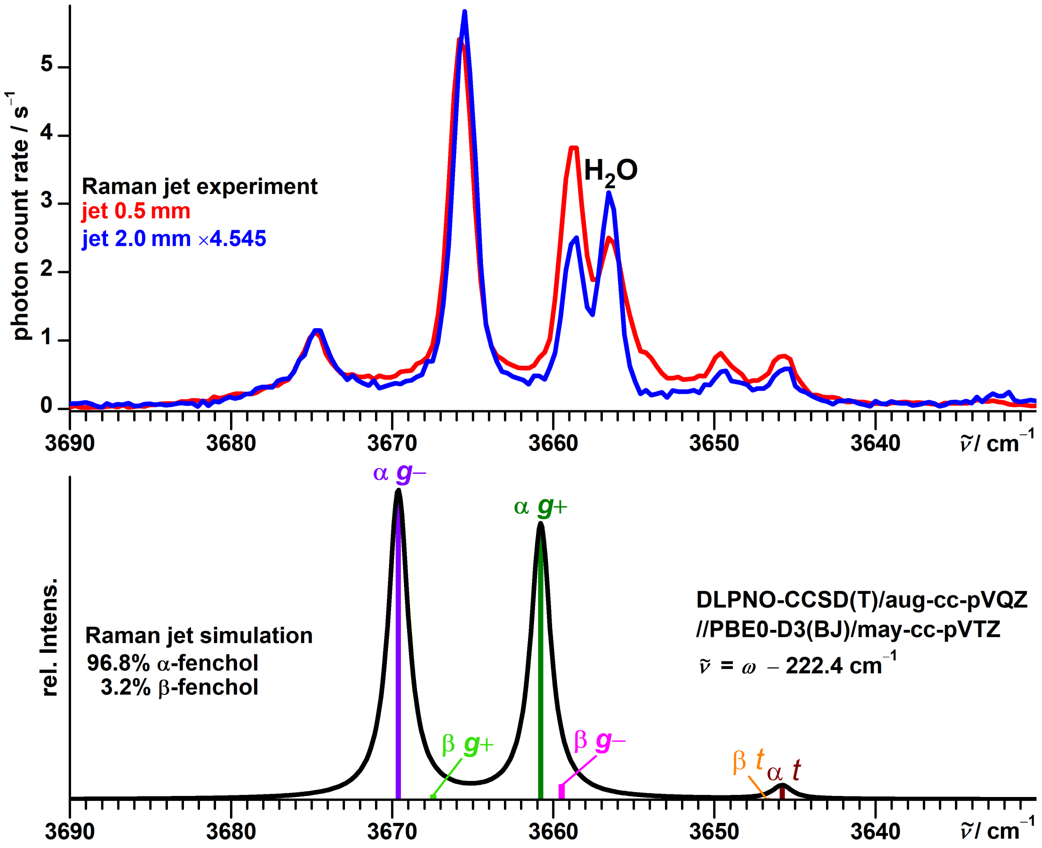

Figure S8: Comparison between experimental and simulated Raman jet spectra of (+)-α-fenchol with (+)-β-fenchol as an assumed impurity. The composition is based on the purity stated by the manufacturer for α-fenchol (96.8%). The simulations assume a Boltzmann distribution of localized conformers at a conformational temperature of 100 K. Harmonic OH stretching wavenumbers are uniformly shifted according to the model based on PBE0 of Ref. 1. As the comparison shows, neither the positions nor the relative intensities can be explained by the mixture of localized conformers of α-fenchol and β-fenchol.

4 Experimental OH Stretch Fundamental Wavenumbers for Alcohol Conformers and Dimer Acceptors Used in the Training Set for the Model for the Isotope Effect

Table S3: Experimental OH stretch fundamental wavenumbers for conformers of protiated and deuterated alcohols as well as dimer acceptors used in the training set for the model for the isotope effect (Figure 7 in the main document). Values marked with an asterisk* were corrected by $-1\,\mathrm{cm}^{-1}$ to account for the calibration error described in Ref. 1.

alcohol	assignment	$\tilde{\nu}_{\mathrm{OH}}$ / cm^{-1}	$\tilde{\nu}_{\mathrm{OD}}$ / cm^{-1}
methanol	monomer	3684[2]	2718*[3]
ethanol	monomer g	3659*[4]	2699*[4]
ethanol	monomer t	3677*[4]	2713*[4]
ethanol	dimer acceptor Xg	3653*[4]	2695*[4]
ethanol	dimer acceptor Xt	3671*[4]	2708*[4]
1-propanol	monomer Gg_{het}	3668*[5]	2705*[5]
1-propanol	monomer Gg_{hom}	3656*[5]	2697*[5]
1-propanol	monomer Gt	3681*[5]	2716*[5]
1-propanol	monomer Tg	3659*[5]	2700*[5]
1-propanol	monomer Tt	3678*[5]	2714*[5]
1-propanol	dimer acceptor a	3674*[5]	2711*[5]
1-propanol	dimer acceptor c	3653*[5]	2695*[5]
2,2,2-trifluoroethanol	monomer g	3656*[3]	2698*[3]
2,2,2-trifluoroethanol	dimer acceptor	3634*[3]	2682*[3]
tert-butyl alcohol	monomer	3642*[3]	2687*[3]
tert-butyl alcohol	dimer acceptor	3631*[3]	2678*[3]

5 Estimated Bond Length Extension Factors

Table S4: Rotational constants B of the hydroxy radical in the electronic ground state but different vibrational states and isotopic compositions.[6,7] From these the relative extension of the bond length r from the equilibrium value is estimated and applied to the calculated distance for the hydroxy group of the alcohols used for the moment of inertia.

quantity	$OH^{\cdot}$	$OD^{\cdot}$
B_e / cm^{-1}	18.871	10.0209
B_0 / cm^{-1}	18.515	9.8831
B_1 / cm^{-1}	17.807	9.6089
$\sqrt{B_e/B_0} \approx r_0/r_e$	1.0096	1.0069
$\sqrt{B_e/B_1} \approx r_1/r_e$	1.0294	1.0212

6 Validation of the 1D Torsion Code for Symmetric Alcohols

6.1 Overview

Table S5: Experimental ground state tunneling splittings Δ(exp) of symmetric alcohols compared to those calculated with the 1D torsion code Δ(calc) based on electronic B3LYP-D3(BJ)/may-cc-pVTZ potentials. Values were rounded to two leading digits for compactness.

alcohol	Δ(exp) / cm^{-1}hc	Δ(calc) / cm^{-1}hc	Δ(calc) / Δ(exp)
methanol CH_3OH	9.1 [8]	9.5	1.05
methanol CH_3OD	2.6 [9]	2.8	1.06
methanol CD_3OH	7.2 [8]	7.3	1.01
methanol CD_3OD	1.5 [10,11]	1.6	1.04
ethanol-OH	3.2 [12]	2.1	0.65
ethanol-OD	$5.7\cdot10^{-1}$ [12]	$3.7\cdot10^{-1}$	0.64
propargyl alcohol-OH	$2.2\cdot10^{+1}$ [13]	$2.3\cdot10^{+1}$	1.07
propargyl alcohol-OD	7.1 [13]	7.1	0.99
2-hydroxyacetonitrile-OH	3.8 [14]	3.9	1.02
2-hydroxyacetonitrile-OD	$5.6\cdot10^{-1}$ [15]	$5.0\cdot10^{-1}$	0.89
4-hydroxy-2-butynenitrile-OH	4.6 [16]	4.8	1.04
4-hydroxy-2-butynenitrile-OD	undetermined	$6.2\cdot10^{-1}$	
allyl alcohol-OH	$4.7\cdot10^{-1}$ [17]	1.0	2.16
allyl alcohol-OD	$3.1\cdot10^{-2}$ [17]	$7.4\cdot10^{-2}$	2.36
methoxymethanol-OH	3.0 [18]	2.4	0.79
methoxymethanol-OD	undetermined	$2.5\cdot10^{-1}$	
fluoromethanol-OH	$5.9\cdot10^{-2}$ [19]	$2.7\cdot10^{-2}$	0.47
fluoromethanol-OD	undetermined	$5.8\cdot10^{-4}$	
2,2,2-trifluoroethanol-OH	$2.0\cdot10^{-1}$ [20]	$2.0\cdot10^{-1}$	1.01
2,2,2-trifluoroethanol-OD	$7.0\cdot10^{-3}$ [20]	$6.2\cdot10^{-3}$	0.90
2-propanol-OH	1.6 [21]	1.4	0.91
2-propanol-OD	$1.5\cdot10^{-1}$ [21]	$1.2\cdot10^{-1}$	0.78
equatorial cyclohexanol-OH	1.7 [22]	1.7	0.98
equatorial cyclohexanol-OD	undetermined	$1.2\cdot10^{-1}$	
cyclopropanol-OH	$1.4\cdot10^{-1}$ [23]	$1.1\cdot10^{-1}$	0.81
cyclopropanol-OD	$5.5\cdot10^{-3}$ [23]	$3.5\cdot10^{-3}$	0.64
tert-butyl alcohol-OH	3.8 [24]	3.6	0.96
tert-butyl alcohol-OD	undetermined	$5.5\cdot10^{-1}$	
axial 1-methylcycloexanol-OH	undetermined	2.9	
axial 1-methylcycloexanol-OD	$5.2\cdot10^{-1}$ [25]	$2.9\cdot10^{-1}$	0.55
equatorial 1-methylcycloexanol-OH	undetermined	3.4	
equatorial 1-methylcycloexanol-OD	$6.1\cdot10^{-1}$ [25]	$4.0\cdot10^{-1}$	0.66
1-vinylcyclopropanol-OH	$7.6\cdot10^{-2}$ [26]	$1.5\cdot10^{-1}$	1.97
1-vinylcyclopropanol-OD	$2.4\cdot10^{-3}$ [26]	$4.3\cdot10^{-3}$	1.76

6.2 Methanol

6.2.1 Methanol CH_3OH/D

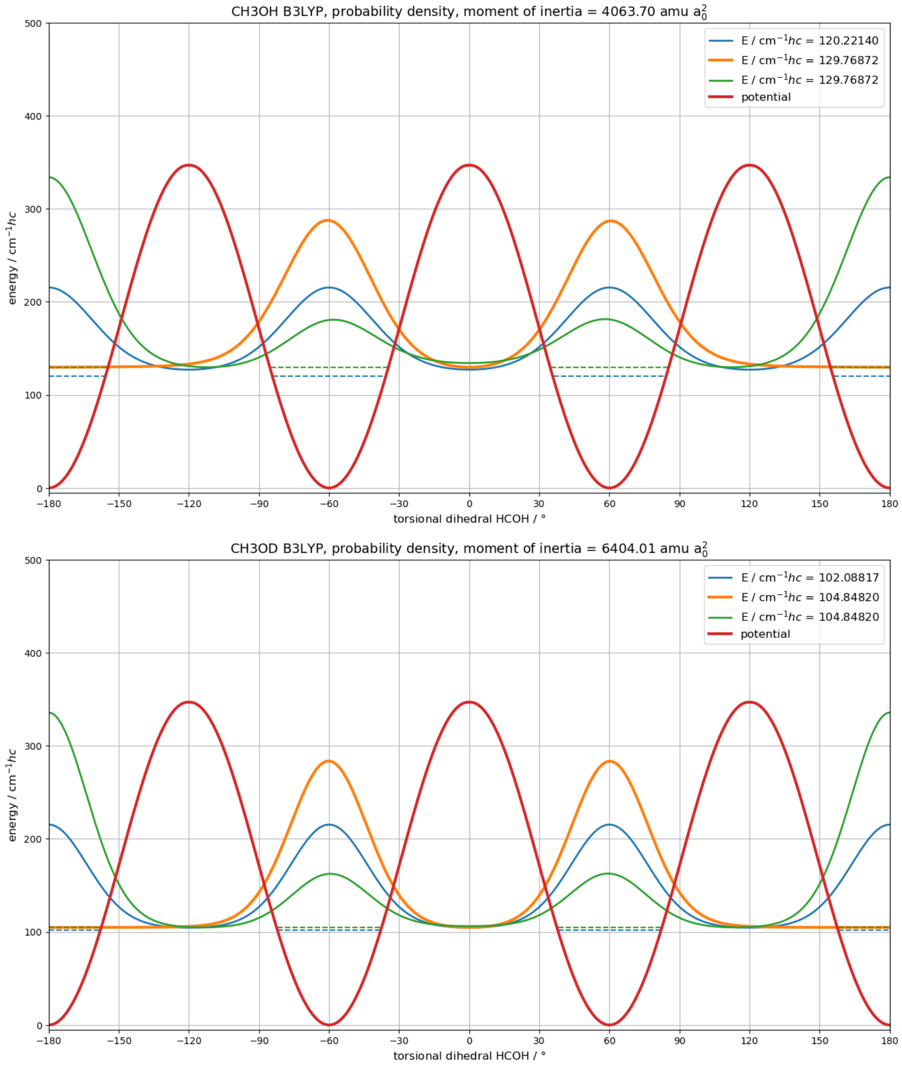

Figure S9: Electronic torsional potential (red trace), calculated at B3LYP-D3(BJ)/may-cc-pVTZ level, with the three lowest torsional states for methanol CH_3OH (top) and CH_3OD (bottom). A constant moment of inertia, based on the minimum geometry, was used.

6.2.2 Methanol CD_3OH/D

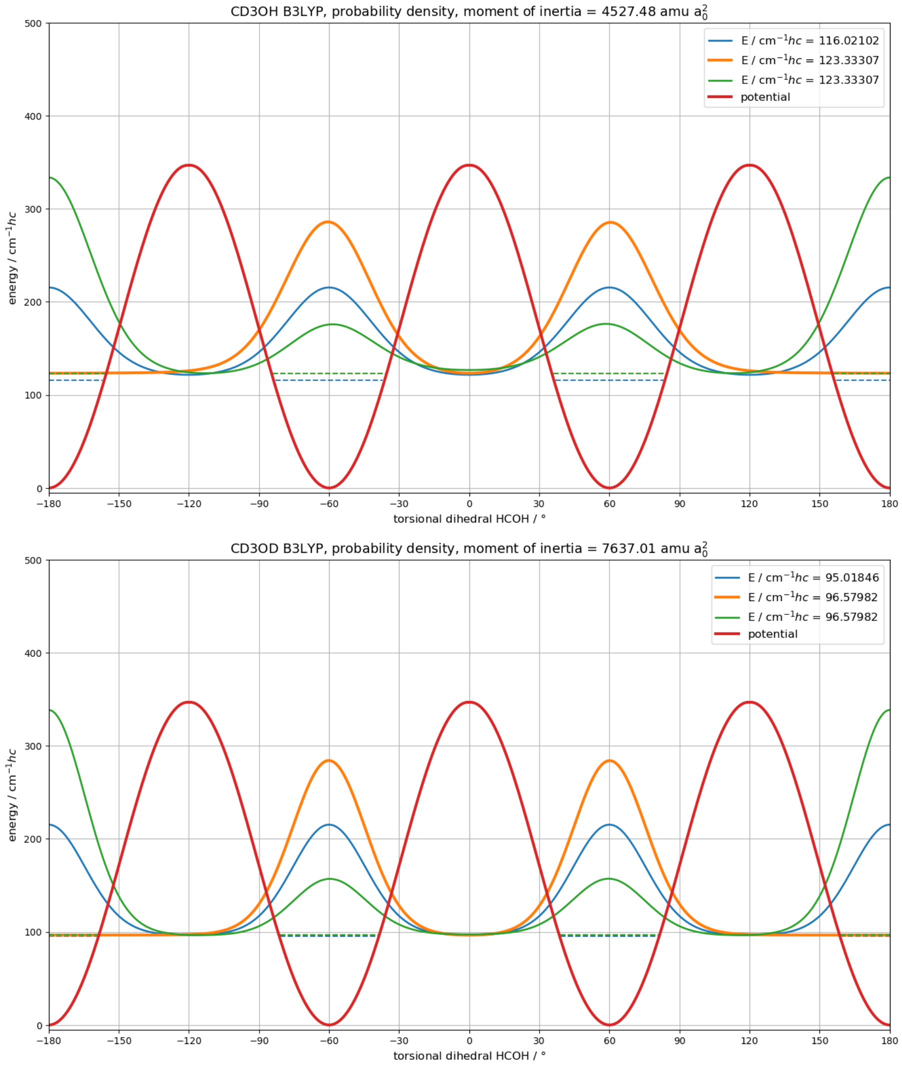

Figure S10: Electronic torsional potential (red trace), calculated at B3LYP-D3(BJ)/may-cc-pVTZ level, with the three lowest torsional states for methanol CD_3OH (top) and CD_3OD (bottom). A constant moment of inertia, based on the minimum geometry, was used.

6.3 Primary Alcohols

6.3.1 Ethanol

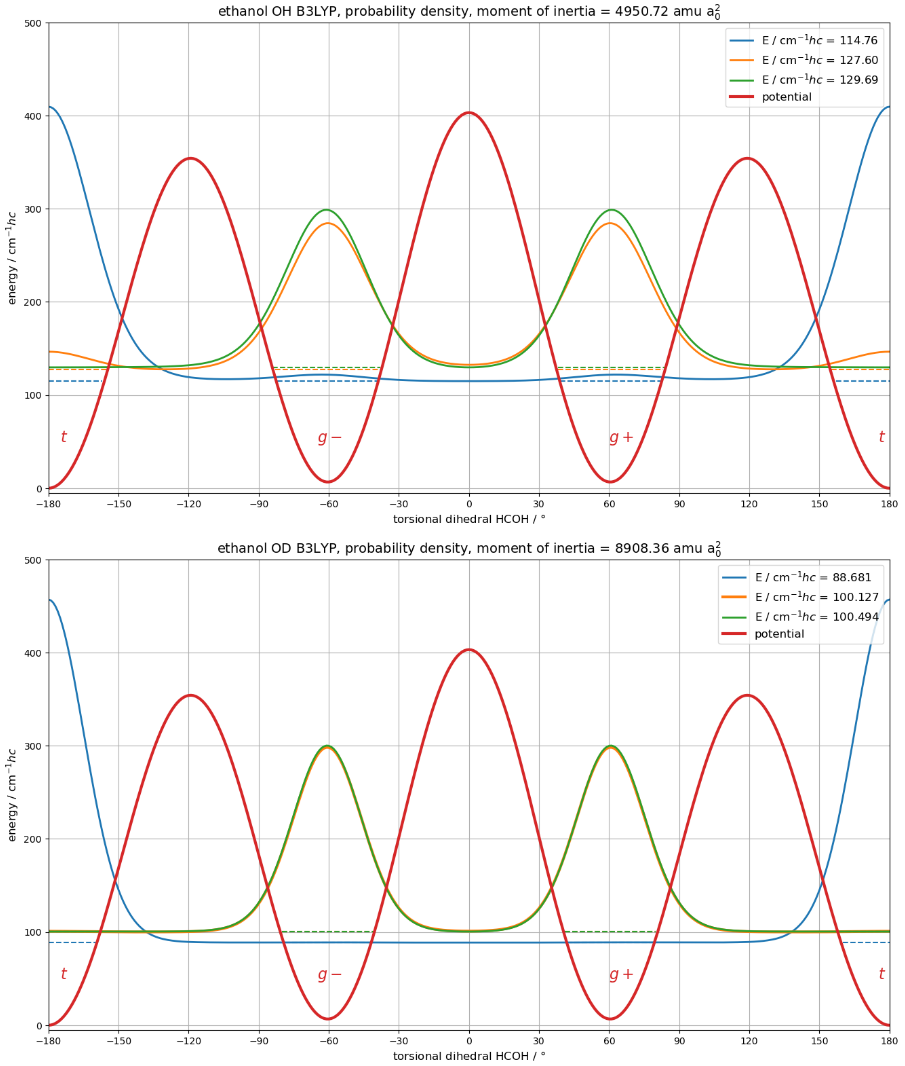

Figure S11: Electronic torsional potential (red trace), calculated at B3LYP-D3(BJ)/may-cc-pVTZ level, with the three lowest torsional states for Ethanol-OH (top) and -OD (bottom). A constant moment of inertia, based on the g minimum geometry, was used.

6.3.2 Propargyl Alcohol

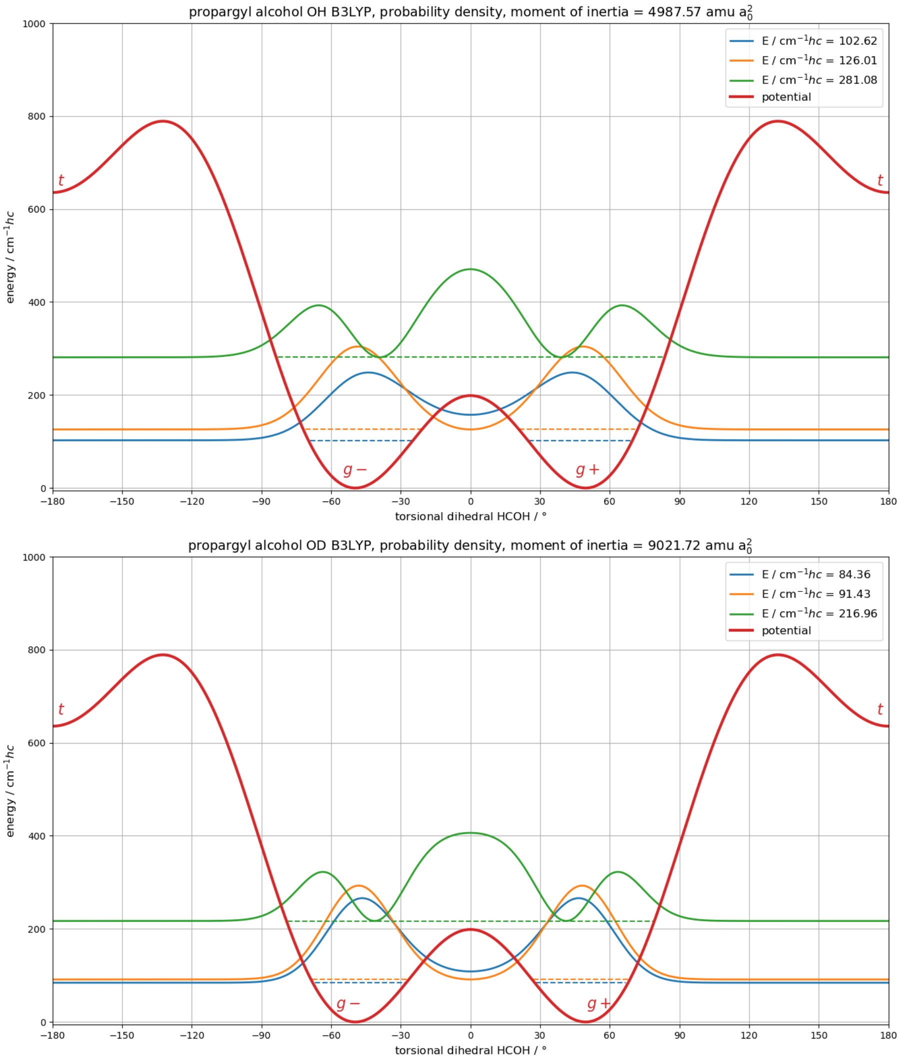

Figure S12: Electronic torsional potential (red trace), calculated at B3LYP-D3(BJ)/may-cc-pVTZ level, with the three lowest torsional states for propargyl alcohol-OH (top) and -OD (bottom). A constant moment of inertia, based on the *g* minimum geometry, was used.

6.3.3 2-Hydroxyacetonitrile

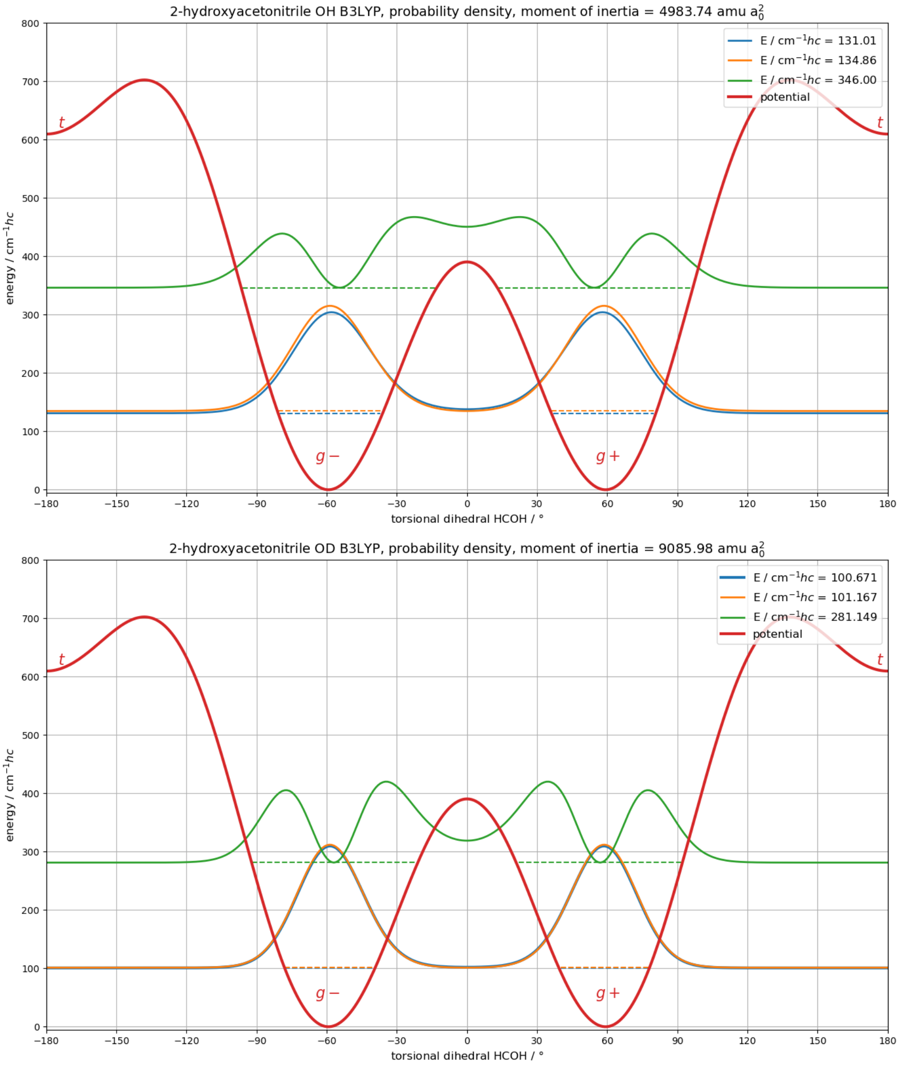

Figure S13: Electronic torsional potential (red trace), calculated at B3LYP-D3(BJ)/may-cc-pVTZ level, with the three lowest torsional states for 2-hydroxyacetonitrile-OH (top) and -OD (bottom). A constant moment of inertia, based on the g minimum geometry, was used.

6.3.4 4-Hydroxy-2-butynenitrile

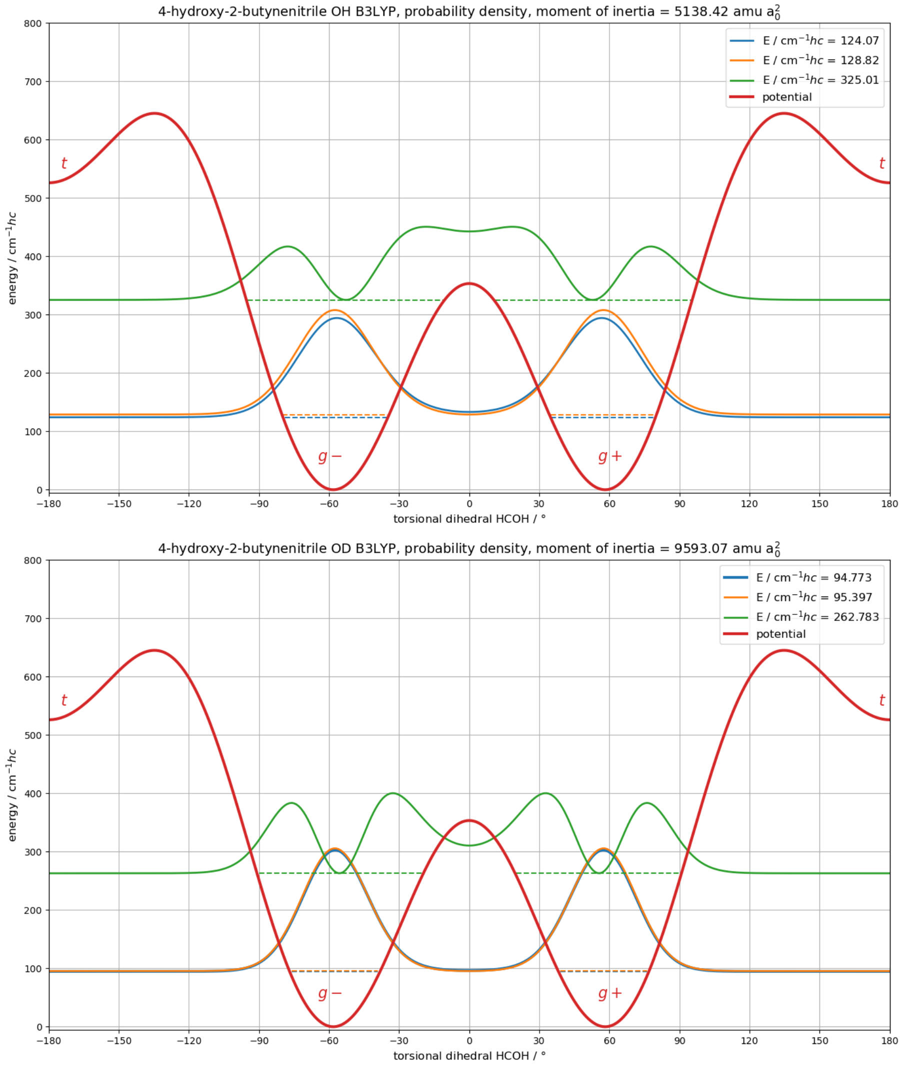

Figure S14: Electronic torsional potential (red trace), calculated at B3LYP-D3(BJ)/may-cc-pVTZ level, with the three lowest torsional states for 4-hydroxy-2-butynenitrile-OH (top) and -OD (bottom). A constant moment of inertia, based on the *g* minimum geometry, was used.

6.3.5 Allyl Alcohol

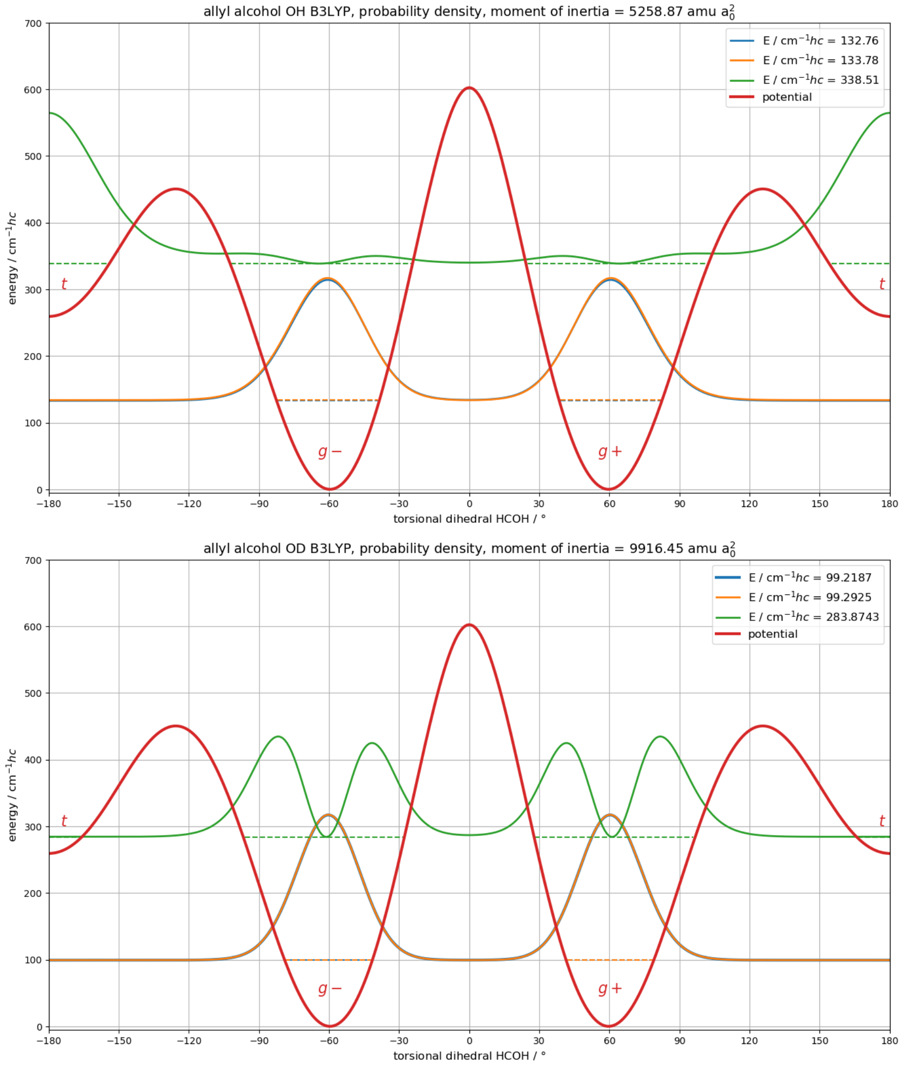

Figure S15: Electronic torsional potential (red trace), calculated at B3LYP-D3(BJ)/may-cc-pVTZ level, with the three lowest torsional states for allyl alcohol-OH (top) and -OD (bottom). A constant moment of inertia, based on the *g* minimum geometry, was used.

6.3.6 Methoxymethanol

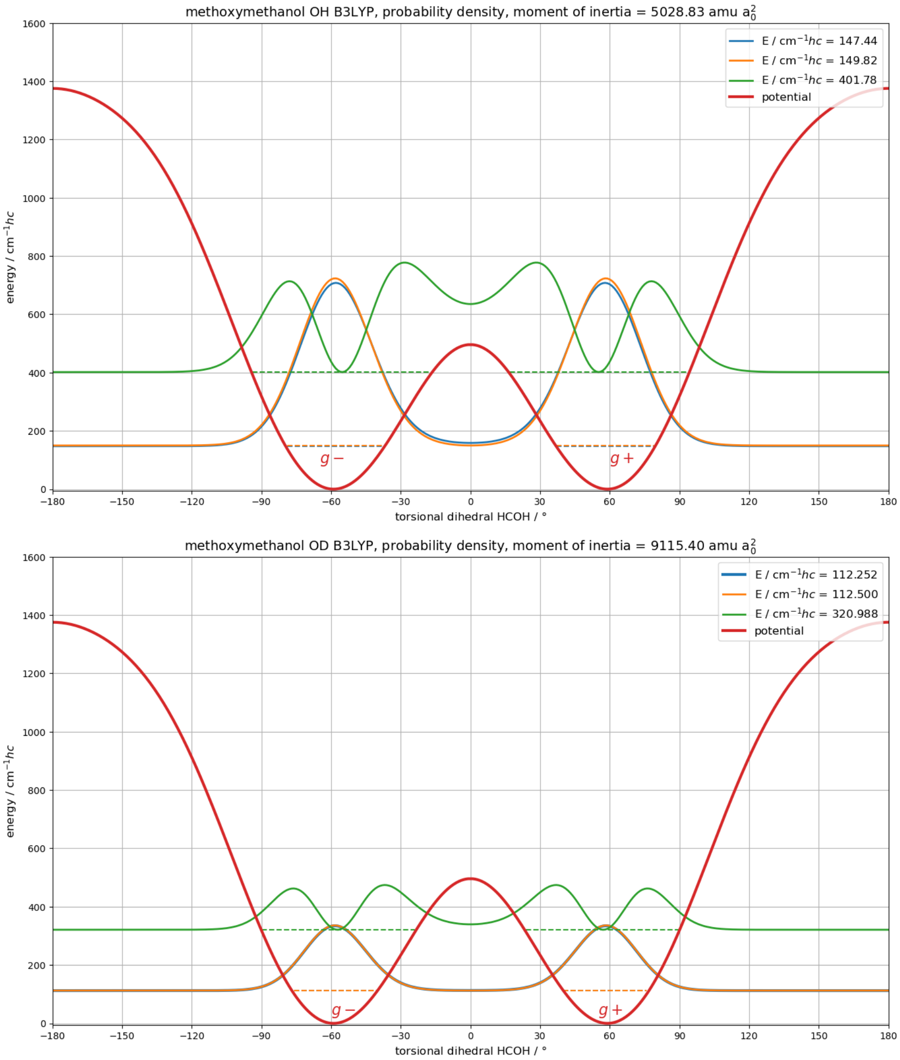

Figure S16: Electronic torsional potential (red trace), calculated at B3LYP-D3(BJ)/may-cc-pVTZ level, with the three lowest torsional states for methoxymethanol-OH (top) and -OD (bottom). A constant moment of inertia, based on the *g* minimum geometry, was used.

6.3.7 Fluoromethanol

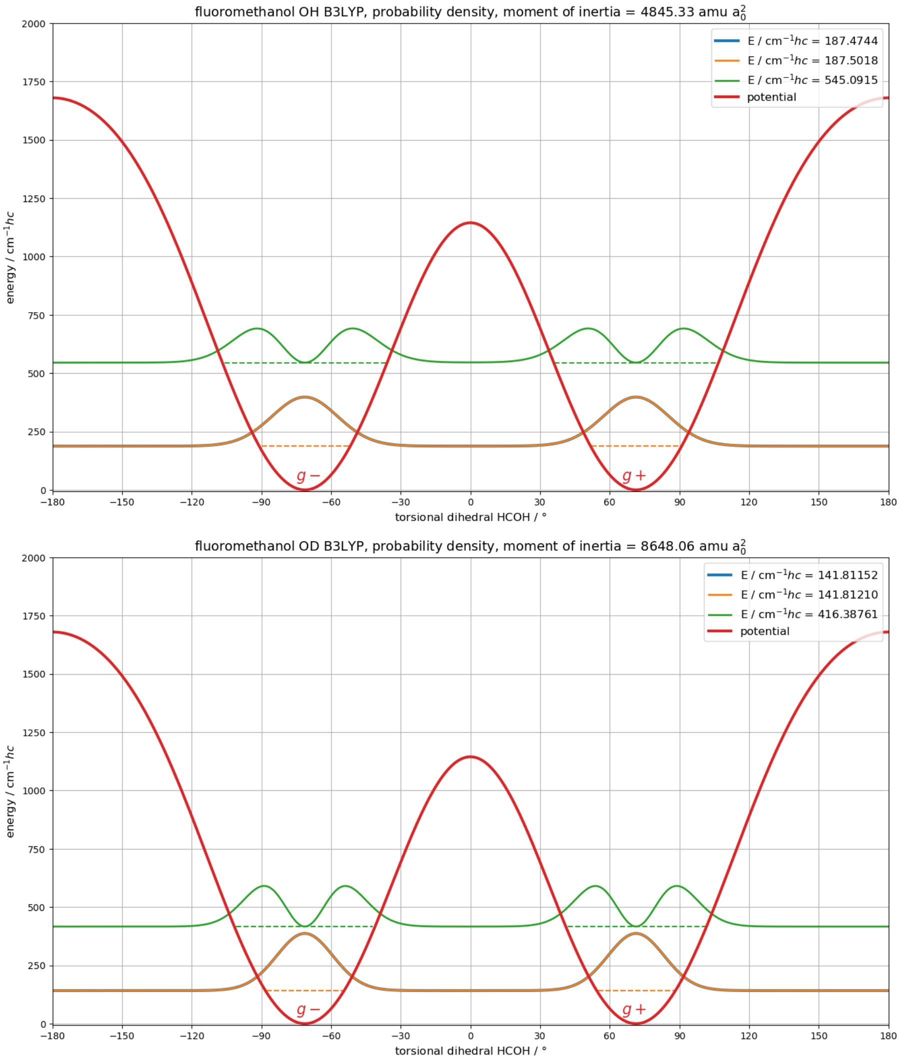

Figure S17: Electronic torsional potential (red trace), calculated at B3LYP-D3(BJ)/may-cc-pVTZ level, with the three lowest torsional states for fluoromethanol-OH (top) and -OD (bottom). A constant moment of inertia, based on the *g* minimum geometry, was used.

6.3.8 2,2,2-Trifluoroethanol

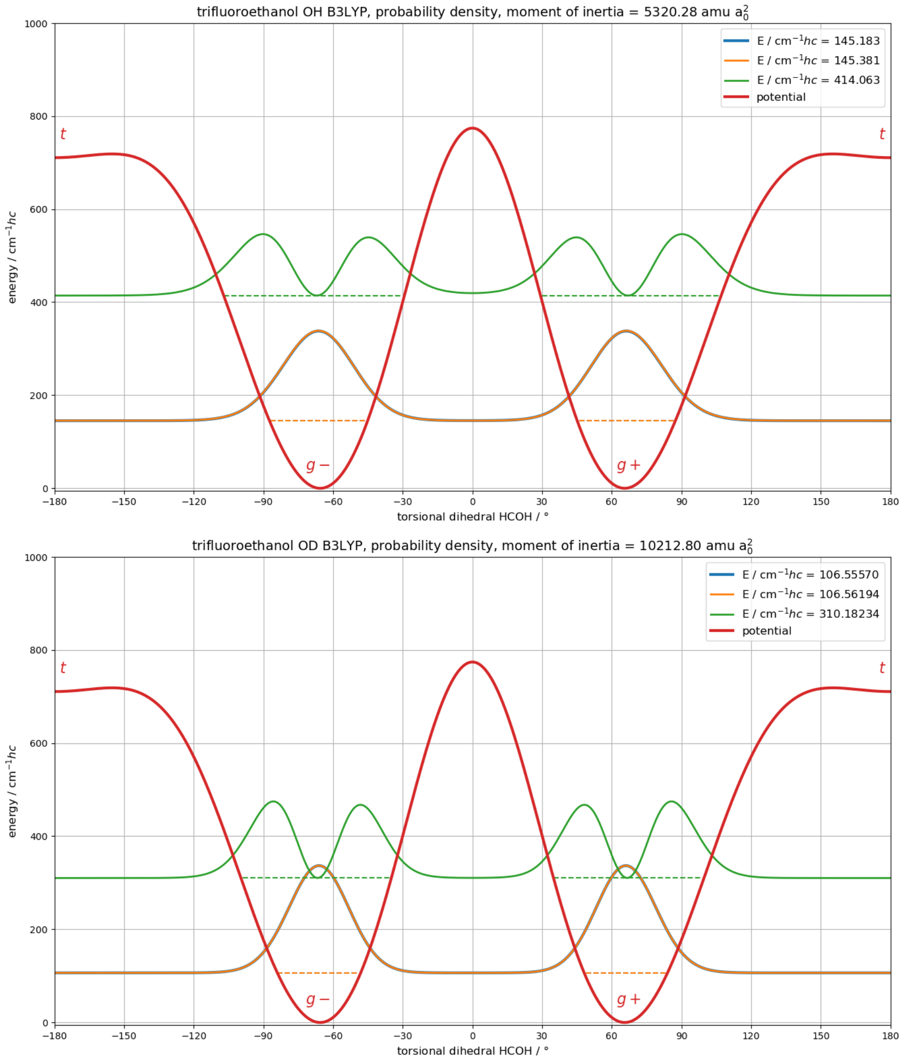

Figure S18: Electronic torsional potential (red trace), calculated at B3LYP-D3(BJ)/may-cc-pVTZ level, with the three lowest torsional states for 2,2,2-trifluoroethanol-OH (top) and -OD (bottom). A constant moment of inertia, based on the g minimum geometry, was used.

6.4 Secondary Alcohols

6.4.1 2-Propanol

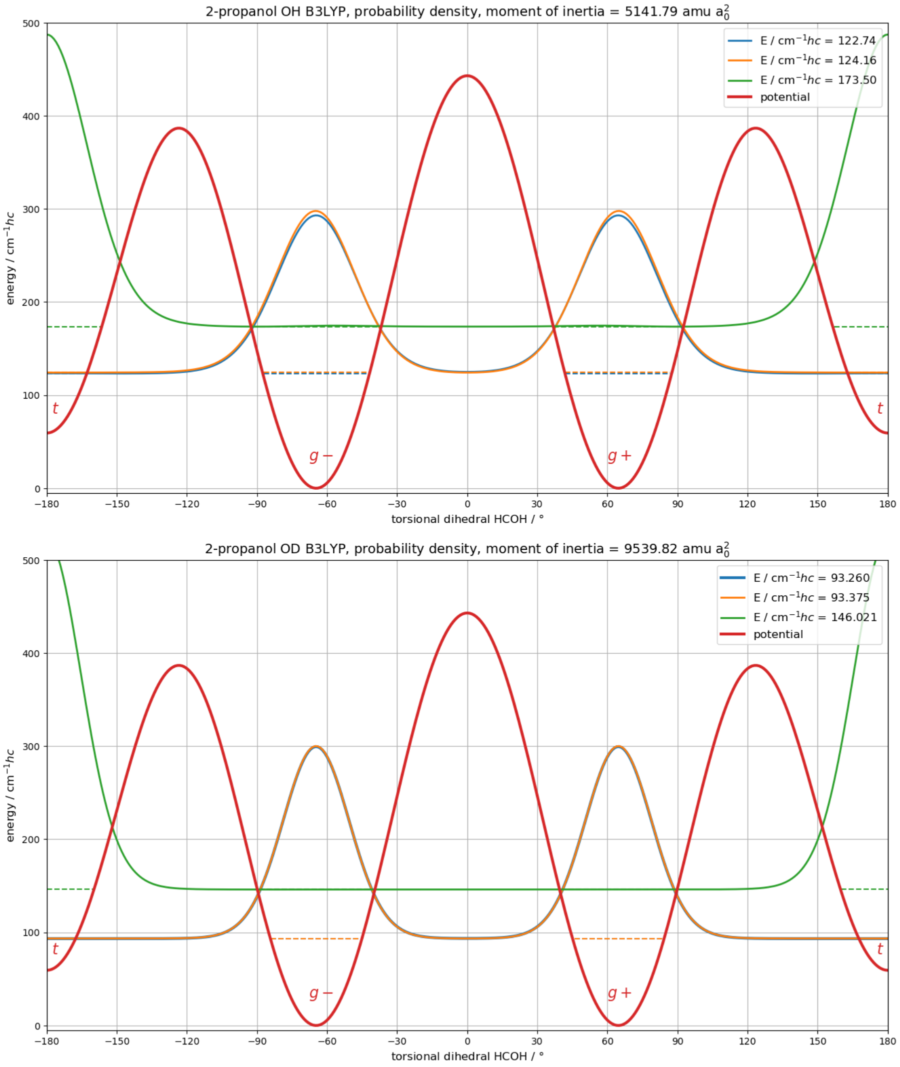

Figure S19: Electronic torsional potential (red trace), calculated at B3LYP-D3(BJ)/may-cc-pVTZ level, with the three lowest torsional states for 2-propanol-OH (top) and -OD (bottom). A constant moment of inertia, based on the *g* minimum geometry, was used.

6.4.2 Equatorial Cyclohexanol

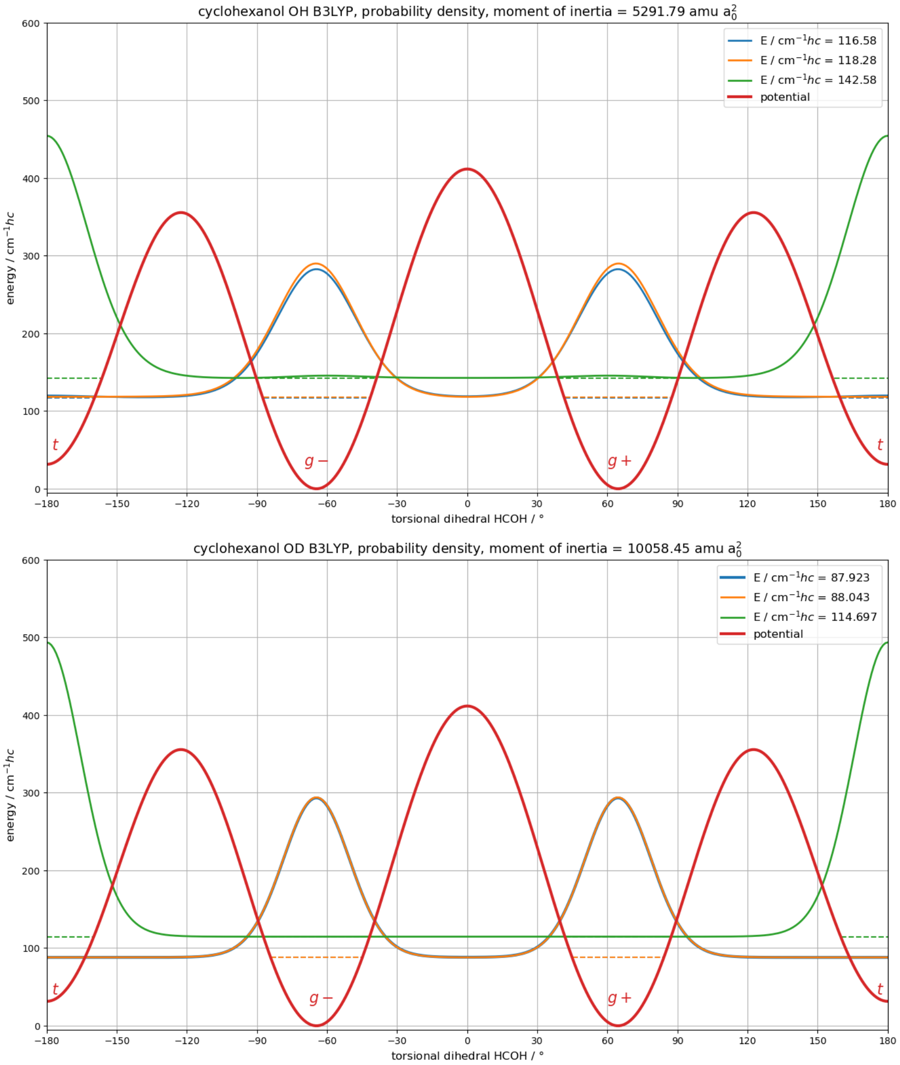

Figure S20: Electronic torsional potential (red trace), calculated at B3LYP-D3(BJ)/may-cc-pVTZ level, with the three lowest torsional states for equatorial cyclohexanol-OH (top) and -OD (bottom). A constant moment of inertia, based on the g minimum geometry, was used.

6.4.3 Cyclopropanol

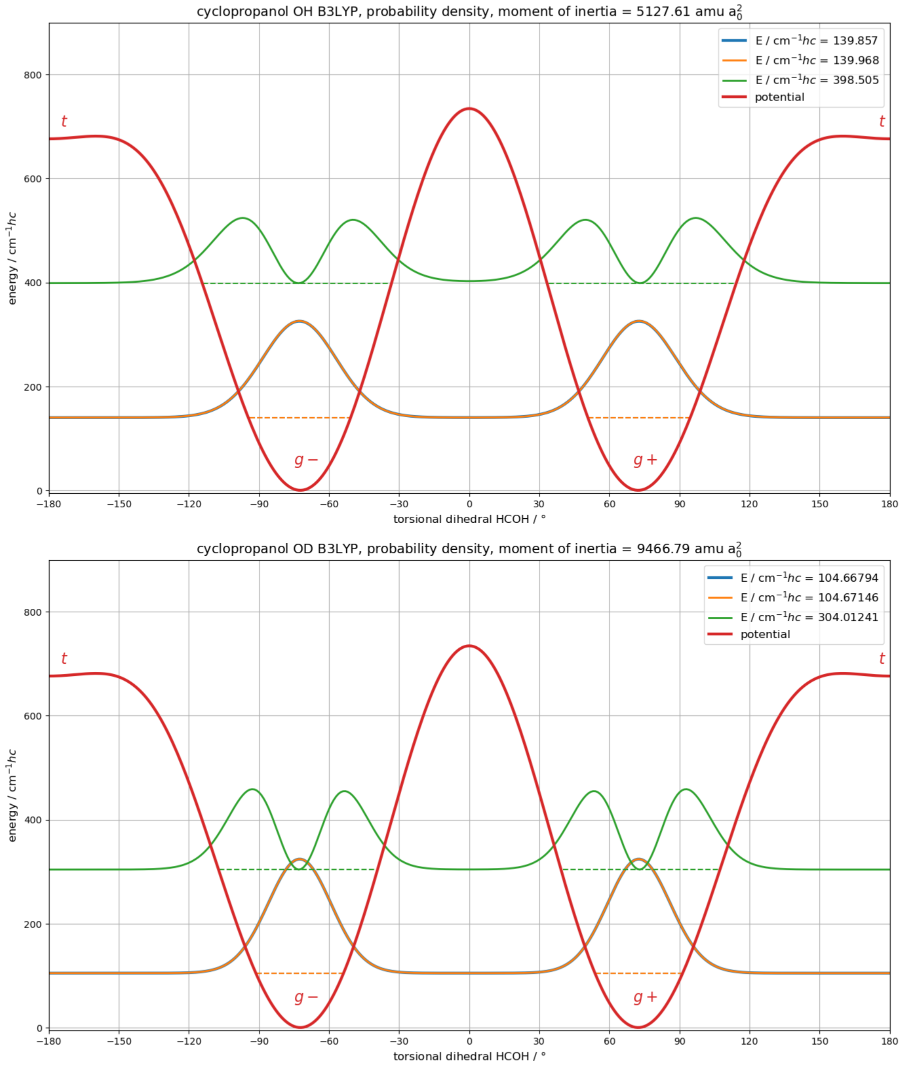

Figure S21: Electronic torsional potential (red trace), calculated at B3LYP-D3(BJ)/may-cc-pVTZ level, with the three lowest torsional states for cyclopropanol-OH (top) and -OD (bottom). A constant moment of inertia, based on the g minimum geometry, was used.

6.5 Tertiary Alcohols

6.5.1 *tert*-Butyl Alcohol

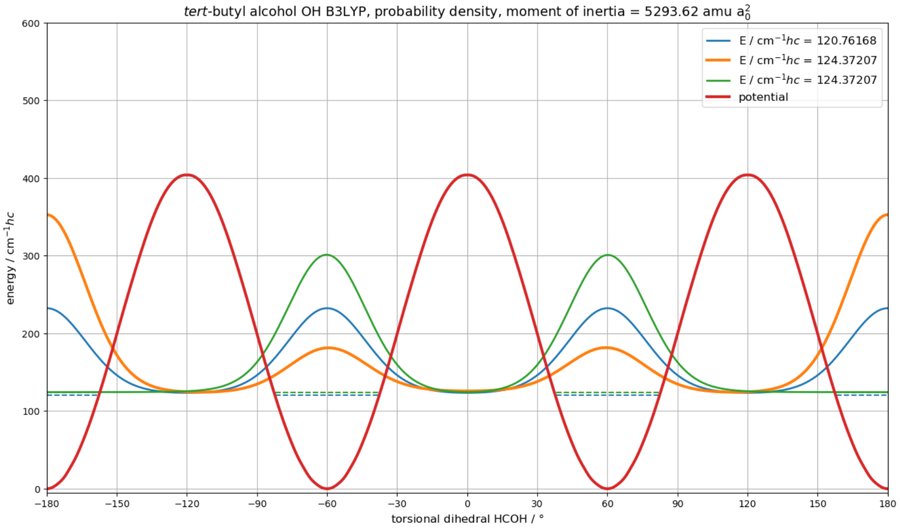

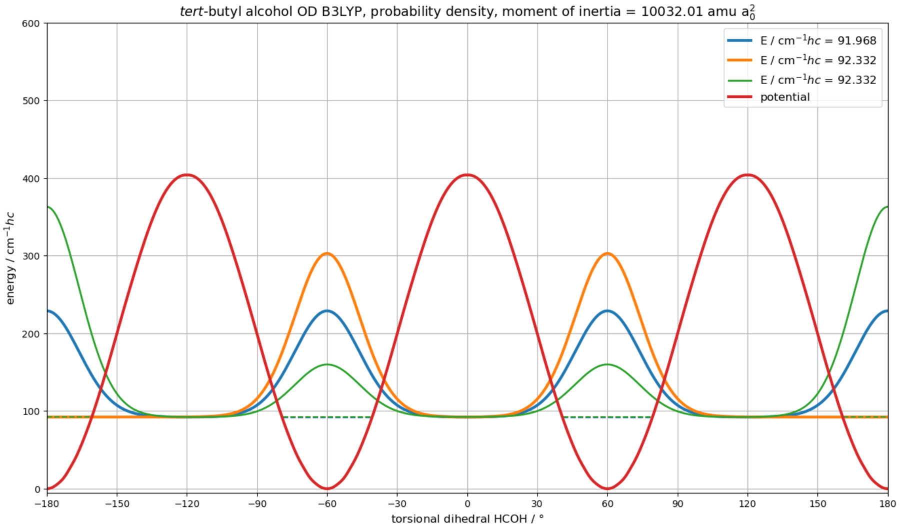

Figure S22: Electronic torsional potential (red trace), calculated at B3LYP-D3(BJ)/may-cc-pVTZ level, with the three lowest torsional states for *tert*-butyl alcohol-OH (top) and -OD (bottom). A constant moment of inertia, based on the *g* minimum geometry, was used.

6.5.2 Axial 1-Methylcyclohexanol

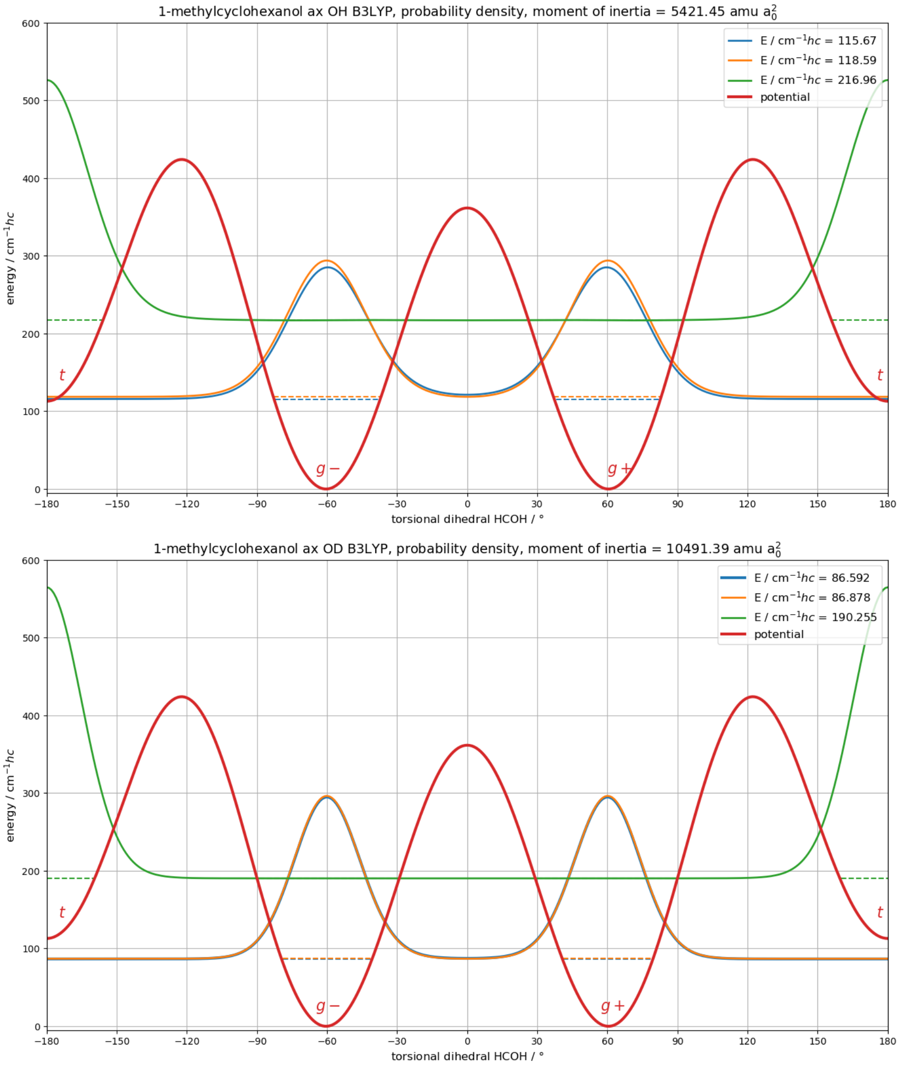

Figure S23: Electronic torsional potential (red trace), calculated at B3LYP-D3(BJ)/may-cc-pVTZ level, with the three lowest torsional states for axial 1-methylcyclohexanol-OH (top) and -OD (bottom). A constant moment of inertia, based on the g minimum geometry, was used.

6.5.3 Equatorial 1-Methylcyclohexanol

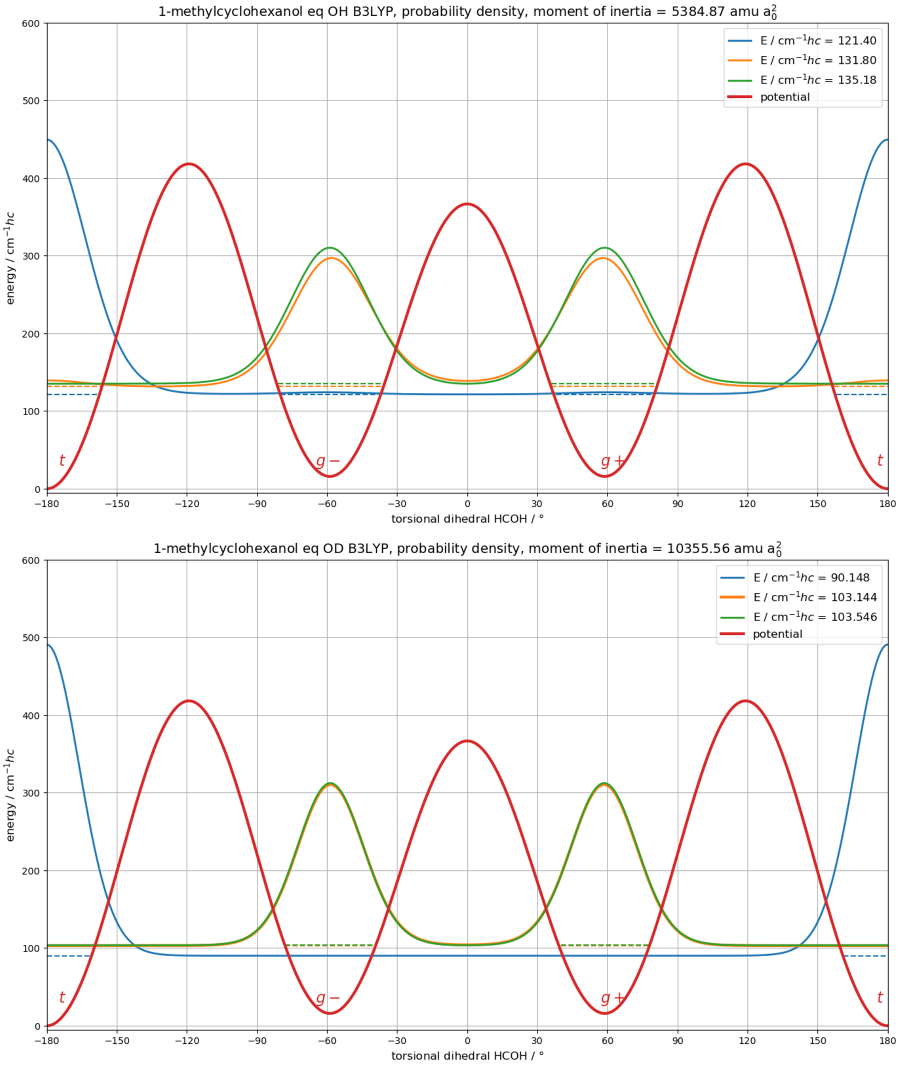

Figure S24: Electronic torsional potential (red trace), calculated at B3LYP-D3(BJ)/may-cc-pVTZ level, with the three lowest torsional states for equatorial 1-methylcyclohexanol-OH (top) and -OD (bottom). A constant moment of inertia, based on the g minimum geometry, was used.

6.5.4 1-Vinylcyclopropanol

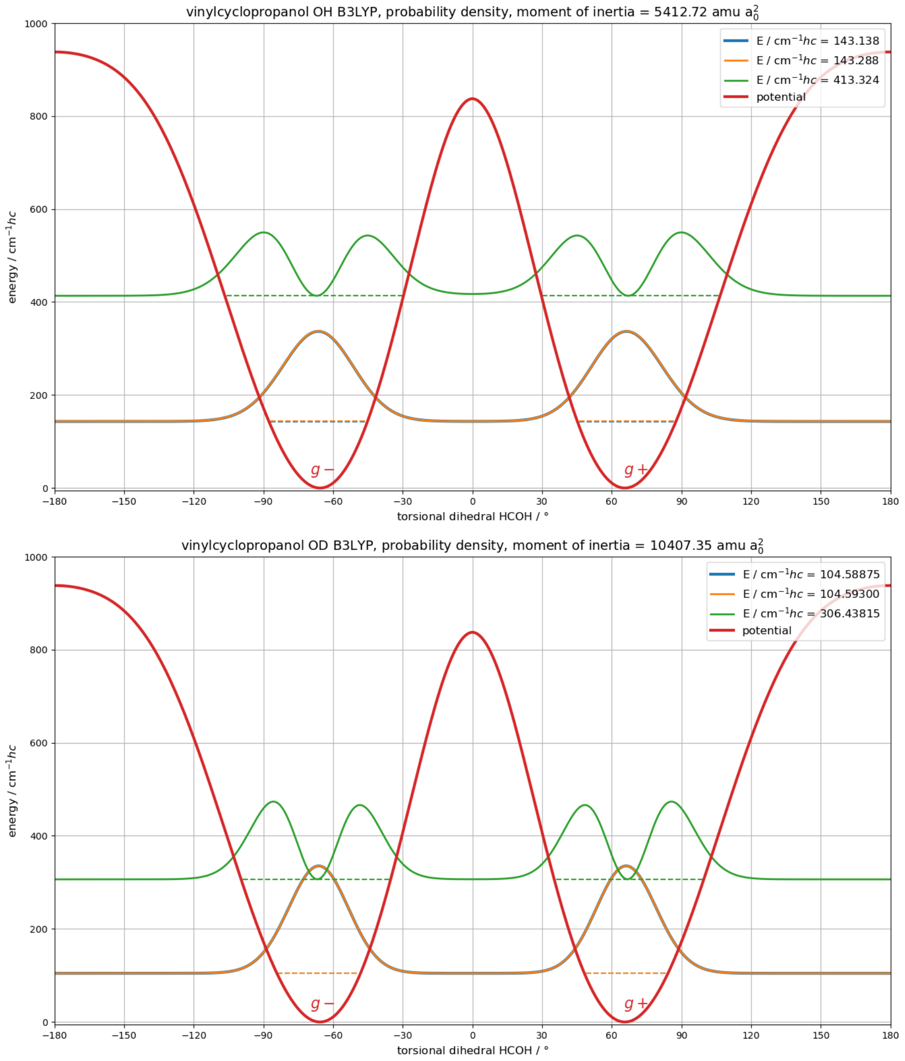

Figure S25: Electronic torsional potential (red trace), calculated at B3LYP-D3(BJ)/may-cc-pVTZ level, with the three lowest torsional states for 1-vinylcyclopropanol-OH (top) and -OD (bottom). A constant moment of inertia, based on the g minimum geometry, was used.

7 Torsional Modelling of (+)-α-Fenchol

7.1 Calculated Properties of the Stationary Points of the Torsional Potential for (+)-α-Fenchol

Table S6: Calculated properties of the stationary points for the torsional potential of (+)-α-fenchol at DLPNO-CCSD(T)/aug-cc-pVQZ//B3LYP-D3(BJ)/may-cc-pVTZ level. τ(HCOH) is the torsional dihedral angle, ω_τ the harmonic torsional wavenumber (for protiated $g+$ and t estimated by averaging of two normal modes with similar torsional character), ω_S the harmonic OH or OD stretching wavenumber, E_{el} the relative electronic energy at B3LYP or CCSD(T) level, E_{ad} the relative energy after harmonic zero-point correction excluding the torsional mode, E_0 the relative energy after full harmonic zero-point correction, I the moment of inertia regarding internal rotation (before extension as described in section 5).

quantity	$g-$	$g+$	t	$g-/g+$	$g-/t$	$g+/t$
τ(HCOH) / °	−52	+52	±180	+1	−118	+117
ω_τ(OH) / cm^{-1}	241	255	252	230i	258i	310i
ω_τ(OD) / cm^{-1}	172	183	171	173i	192i	230i
ω_S(OH) / cm^{-1}	3837	3829	3820	3878	3851	3838
ω_S(OD) / cm^{-1}	2794	2787	2779	2824	2803	2794
E_{el}^{B3LYP} / cm^{-1}hc	0	13	52	213	383	526
$E_{el}^{CCSD(T)}$ / cm^{-1}hc	0	−4	111	206	407	556
$E_{ad\ (OH)}^{CCSD(T)}$ / cm^{-1}hc	0	3	122	233	440	567
$E_{ad\ (OD)}^{CCSD(T)}$ / cm^{-1}hc	0	3	127	219	428	558
$E_{0\ (OH)}^{CCSD(T)}$ / cm^{-1}hc	0	10	127	112	320	446
$E_{0\ (OD)}^{CCSD(T)}$ / cm^{-1}hc	0	9	126	133	343	472
I(OH) / amu a$_0^2$	5272	5323	5303	5246	5219	5258
I(OD) / amu a$_0^2$	10288	10390	10414	10252	10207	10284

7.2 Torsional Potentials for (+)-α-Fenchol

7.2.1 Ground State at B3LYP-D3(BJ) Level

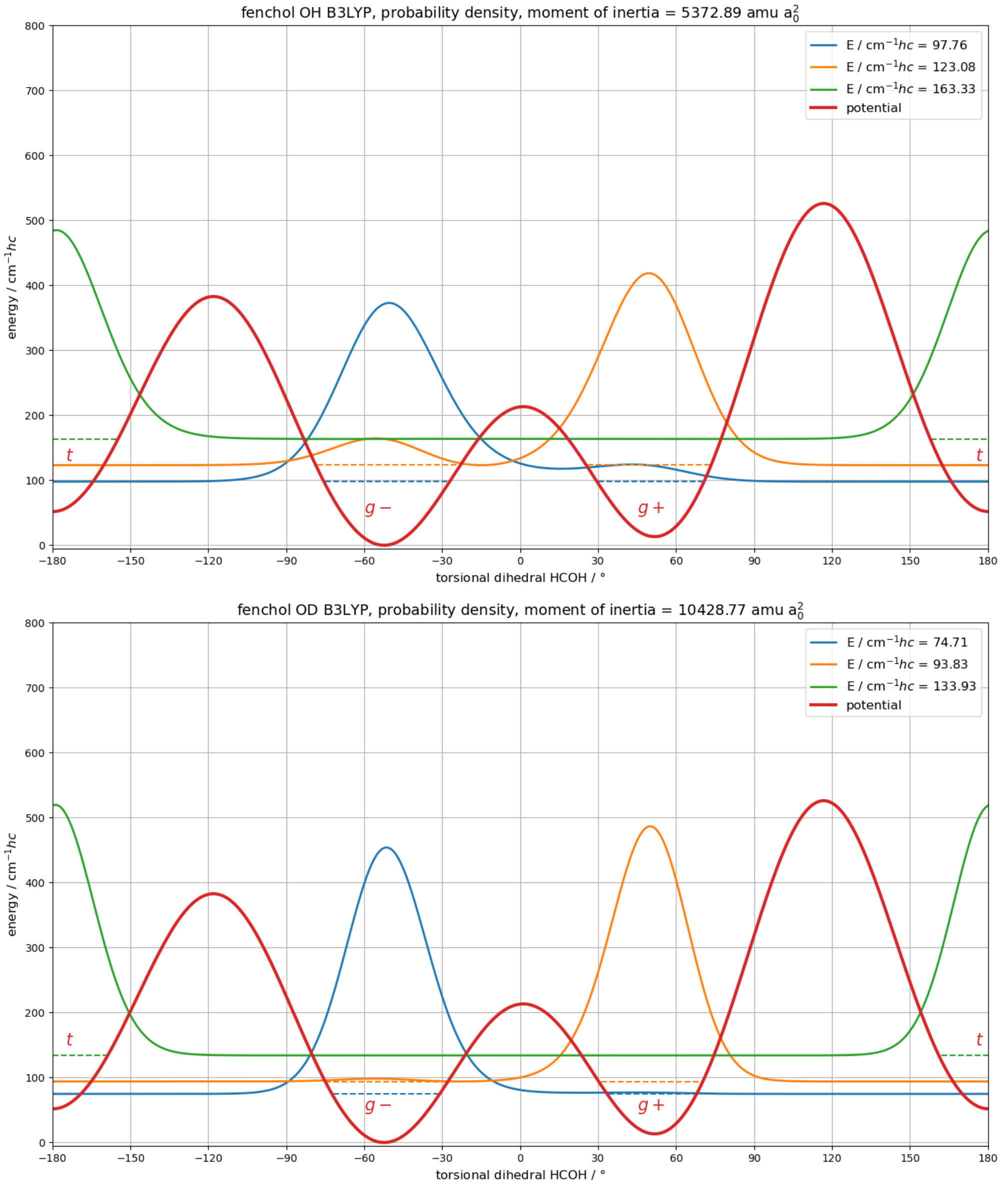

Figure S26: Electronic torsional potential (red trace) of (+)-α-fenchol calculated at B3LYP-D3(BJ)/may-cc-pVTZ level. A constant moment of inertia, based on the $g-$ minimum geometry, was used for the protiated (top) and deuterated alcohol (bottom).

7.2.2 Symmetrized Ground State at B3LYP-D3(BJ) Level

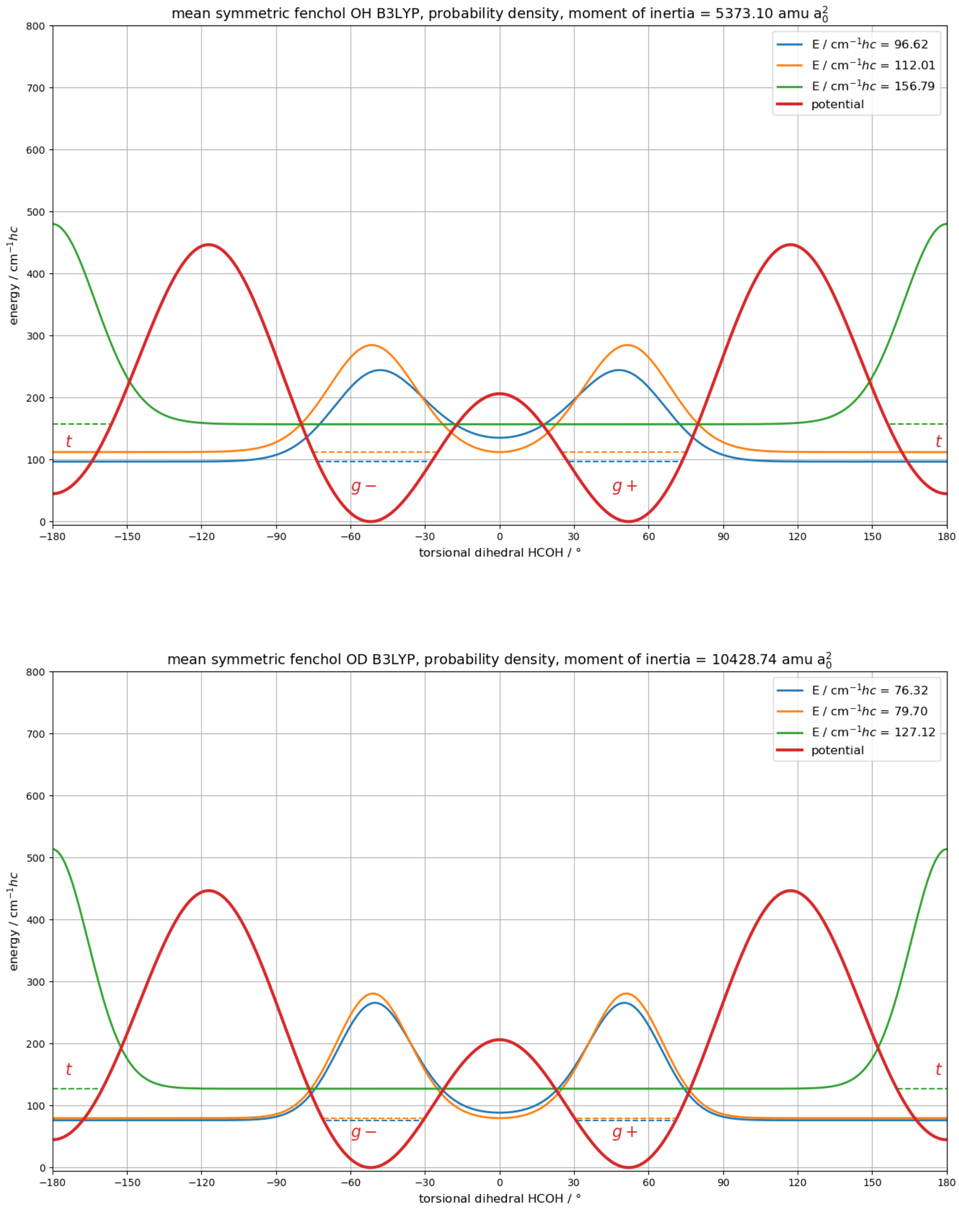

Figure S27: Electronic torsional potential (red trace) of (+)-α-fenchol calculated at B3LYP-D3(BJ)/may-cc-pVTZ level (as in Figure S26) but symmetrized by averaging the two half-potentials separated at the $g-/g+$ transition state. A constant moment of inertia, based on the $g-$ minimum geometry, was used for the protiated (top) and deuterated alcohol (bottom).

7.2.3 Symmetrized Ground State at DLPNO-CCSD(T)//B3LYP-D3(BJ) Level

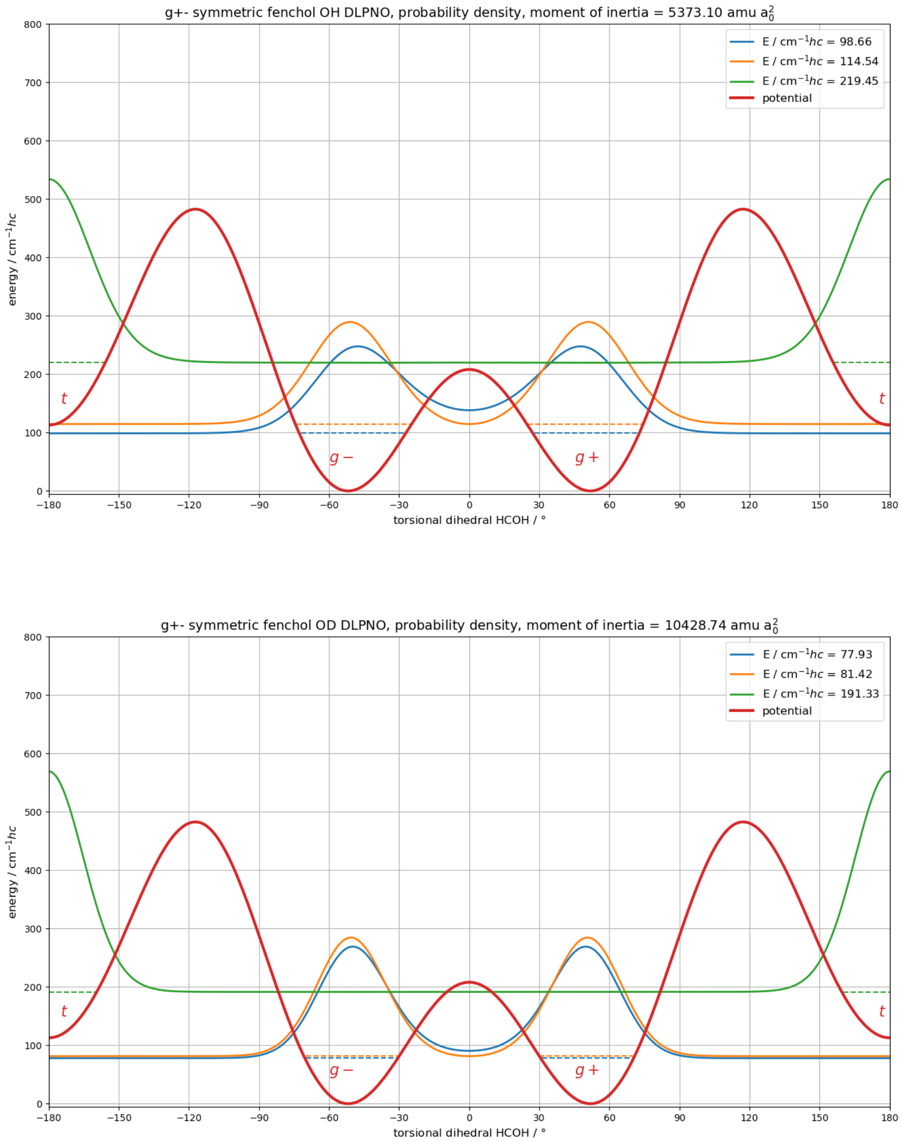

Figure S28: Electronic torsional potential (red trace) of (+)-α-fenchol calculated at B3LYP-D3(BJ)/may-cc-pVTZ level, scaled to DLPNO-CCSD(T)/aug-cc-pVQZ single-point corrections for the six stationary points (as in Figure 10 of the main document) but symmetrized by averaging the two half-potentials separated at the $g-/g+$ transition state. A constant moment of inertia, based on the $g-$ minimum geometry, was used for the protiated (top) and deuterated alcohol (bottom).

7.2.4 Ground State at Zero-point Corrected DLPNO-CCSD(T)//B3LYP-D3(BJ) Level

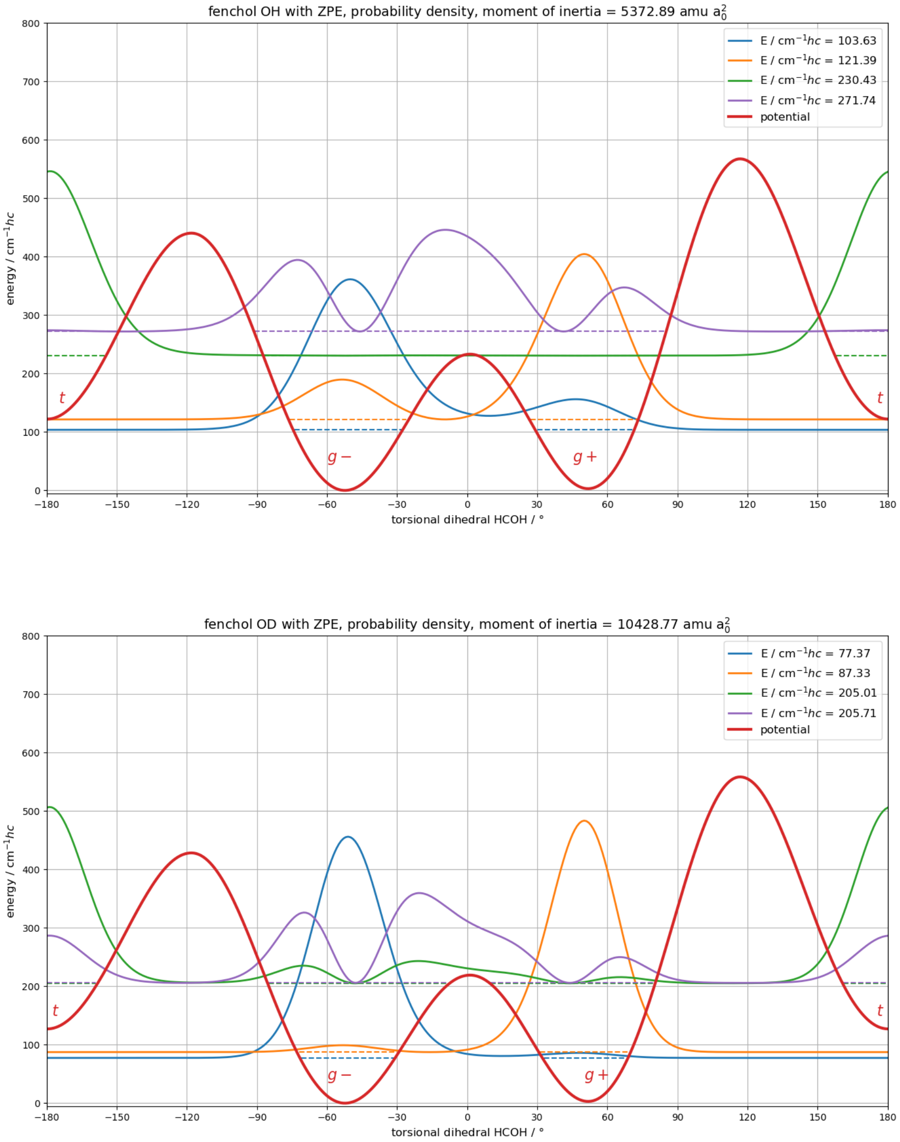

Figure S29: Alternative torsional potential (red trace) of (+)-α-fenchol calculated at B3LYP-D3(BJ)/may-cc-pVTZ level, scaled to DLPNO-CCSD(T)/aug-cc-pVQZ single-point corrections plus (different from Figure 10 in the main document) B3LYP vibrational non-torsional zero-point energy for the six stationary points. A constant moment of inertia, based on the $g-$ minimum geometry, was used for the protiated (top) and deuterated alcohol (bottom). For the latter there is an accidental near-degeneracy also between the third and fourth torsional state leading to weak resonant mixing.

7.2.5 OH/OD Stretch Excited State at DLPNO-CCSD(T)/aug-cc-pVQZ//B3LYP-D3(BJ) Level and Franck-Condon Factors

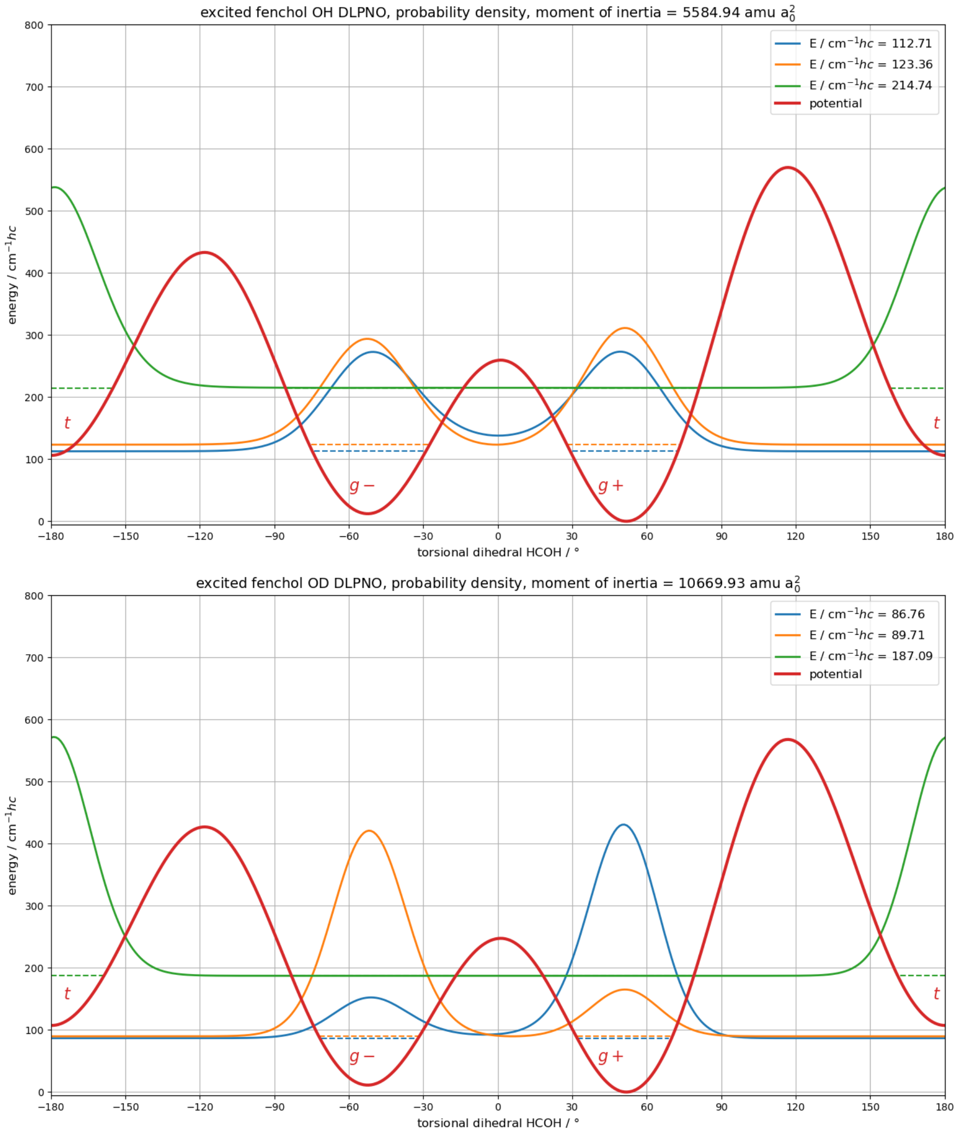

Figure S30: Estimated torsional potential (red trace) of (+)-α-fenchol in the OH (top) or OD (bottom) stretch excited state. Compared to the ground-state DLPNO-CCSD(T)/aug-cc-pVQZ//B3LYP-D3(BJ)/may-cc-pVTZ potential in Figure 10 of the main document, in addition the respective harmonic B3LYP OH or OD stretch wavenumber was added to the six stationary points before scaling.

Table S7: Franck-Condon factors $\langle \chi^1_{m'} \chi^0_m \rangle^2$ for (+)-α-fenchol-OH based on Figure 10 in the main document and Figure S30.

	$\chi^1_1 \mathrel{\widehat{=}} l_1$	$\chi^1_2 \mathrel{\widehat{=}} u_1$	χ^1_3
$\chi^0_1 \mathrel{\widehat{=}} l_0$	0.958	$3.63{\cdot}10^{-2}$	$4.56{\cdot}10^{-6}$
$\chi^0_2 \mathrel{\widehat{=}} u_0$	$3.57{\cdot}10^{-2}$	0.963	$1.43{\cdot}10^{-6}$
χ^0_1	$2.33{\cdot}10^{-5}$	$1.45{\cdot}10^{-6}$	0.914

Table S8: Franck-Condon factors for (+)-α-fenchol-OD based on Figure 10 in the main document and Figure S30.

	$\chi^1_1 \mathrel{\widehat{=}} l_1$	$\chi^1_2 \mathrel{\widehat{=}} u_1$	χ^1_3
$\chi^0_1 \mathrel{\widehat{=}} l_0$	0.487	0.511	$6.70{\cdot}10^{-8}$
$\chi^0_2 \mathrel{\widehat{=}} u_0$	0.510	0.488	$1.63{\cdot}10^{-8}$
χ^0_3	$2.43{\cdot}10^{-6}$	$2.68{\cdot}10^{-7}$	0.899

7.2.6 OH/OD Stretch Excited State at Zero-point Corrected DLPNO-CCSD(T)/aug-cc-pVQZ//B3LYP-D3(BJ) Level and Franck-Condon Factors

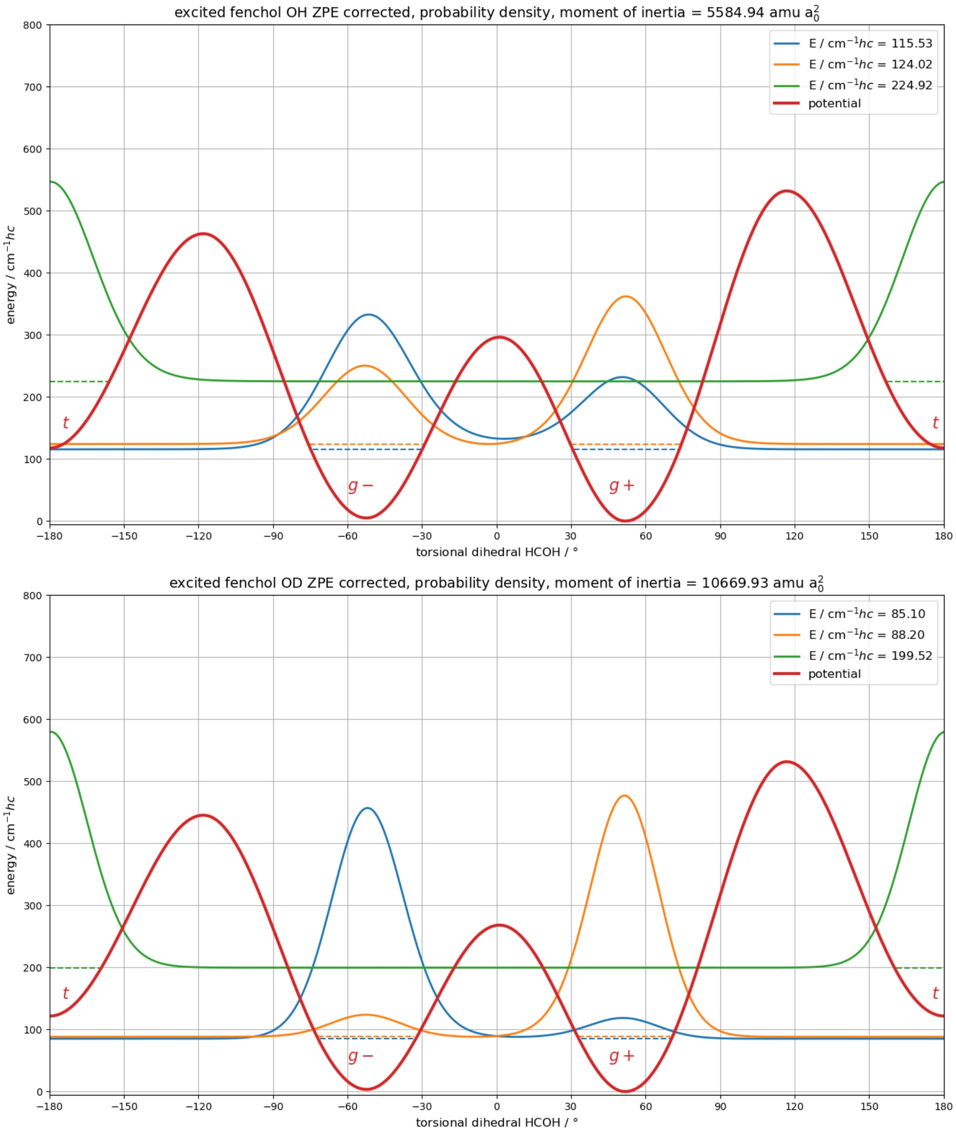

Figure S31: Alternative estimated torsional potential (red trace) of (+)-α-fenchol in the OH (top) or OD (bottom) stretch excited state. Compared with Figure S30, additional non-torsional zero-point corrections at harmonic B3LYP level were added to the stationary points before scaling.

Table S9: Franck-Condon factors $\left\langle \chi^1_{m'} \chi^0_m \right\rangle^2$ for (+)-α-fenchol-OH based on Figures S29 and S31.

	$\chi^1_1 \hat{=} l_1$	$\chi^1_2 \hat{=} u_1$	χ^1_3
$\chi^0_1 \hat{=} l_0$	0.954	$3.81 \cdot 10^{-2}$	$5.63 \cdot 10^{-6}$
$\chi^0_2 \hat{=} u_0$	$3.65 \cdot 10^{-2}$	0.959	$8.44 \cdot 10^{-6}$
χ^0_3	$2.79 \cdot 10^{-5}$	$9.82 \cdot 10^{-6}$	0.914

Table S10: Franck-Condon factors $\left\langle \chi^1_{m'} \chi^0_m \right\rangle^2$ for (+)-α-fenchol-OD based on Figures S29 and S31.

	χ^1_1	χ^1_2	χ^1_3
$\chi^0_1 \hat{=} l_0$	0.979	$1.79 \cdot 10^{-2}$	$1.47 \cdot 10^{-7}$
$\chi^0_2 \hat{=} u_0$	$1.73 \cdot 10^{-2}$	0.978	$1.75 \cdot 10^{-7}$
χ^0_3	$6.74 \cdot 10^{-4}$	$2.75 \cdot 10^{-4}$	0.731

7.2.7 Artificial Localization of the Ground State at DLPNO-CCSD(T)/aug-cc-pVQZ//B3LYP-D3(BJ) Level

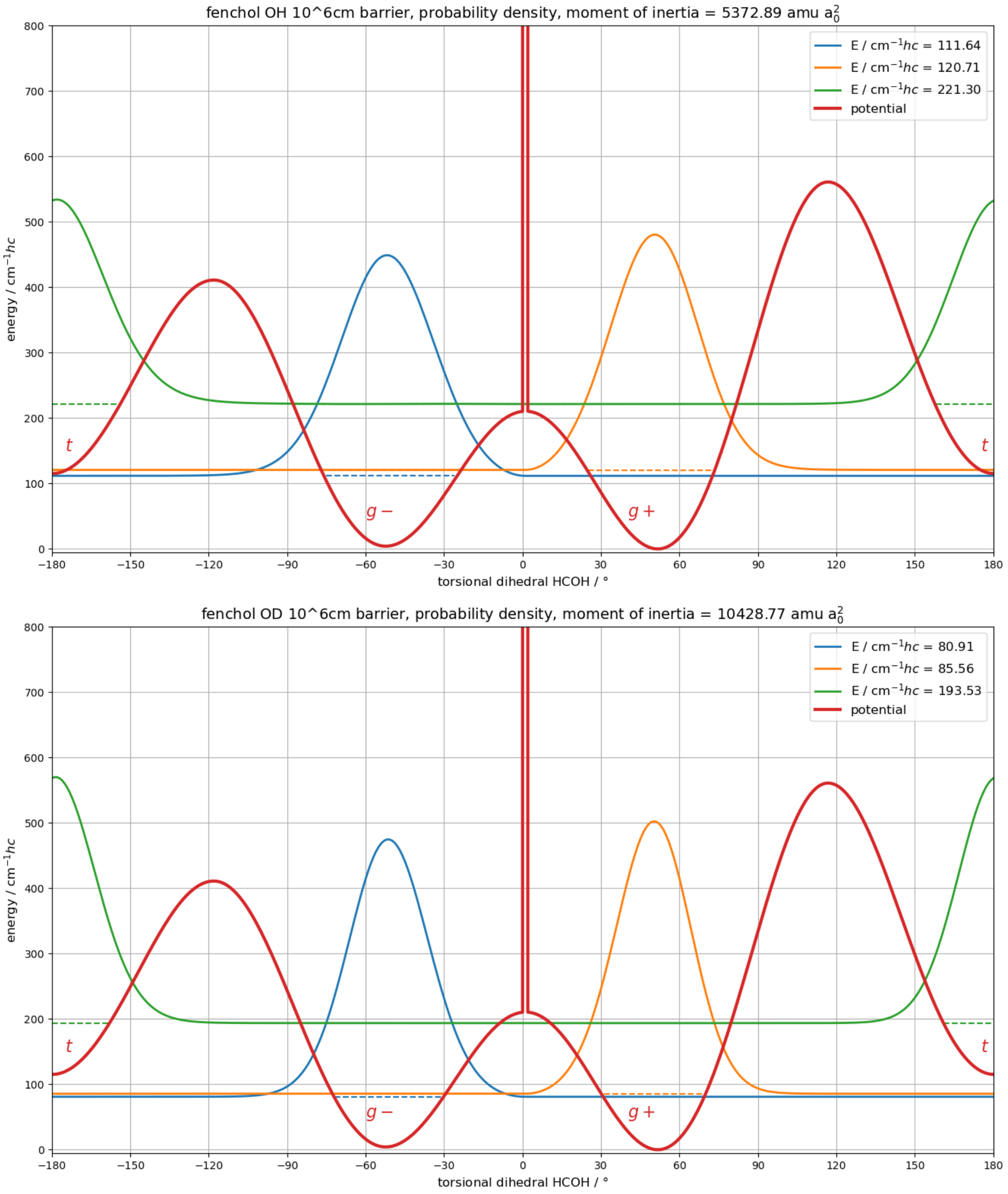

Figure S32: Electronic torsional potential (red trace) of (+)-α-fenchol-OH (top) and -OD (bottom) calculated at B3LYP-D3(BJ)/may-cc-pVTZ level, scaled to DLPNO-CCSD(T)/aug-cc-pVQZ single-point corrections for the six stationary points as in Figure 10 of the main document, but with an additional narrow (1°) and towering ($10^6\,cm^{-1}hc$) rectangle barrier at the $g-/g+$ transition state to suppress tunneling.

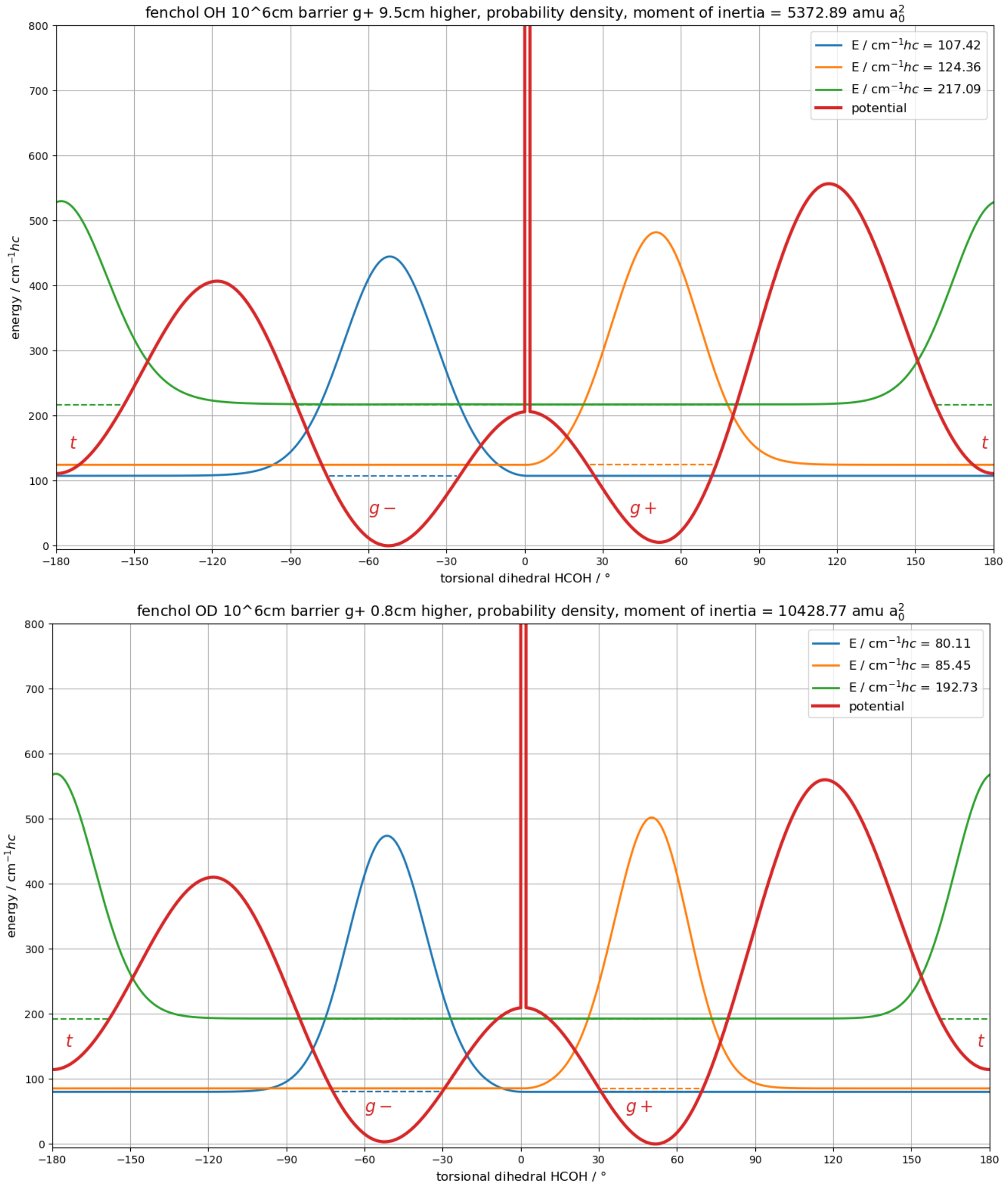

Figure S33: Same as Figure S32 but the $g+$ well has been lifted (by $9.5\,\mathrm{cm}^{-1}hc$ for OH and $0.8\,\mathrm{cm}^{-1}hc$ for OD) to reproduce the same torsional splitting as with tunneling (Figure 10 in the main document).

7.2.8 Artificial Localization of the OH/OD Stretch Excited State at DLPNO-CCSD(T)/aug-cc-pVQZ//B3LYP-D3(BJ) Level

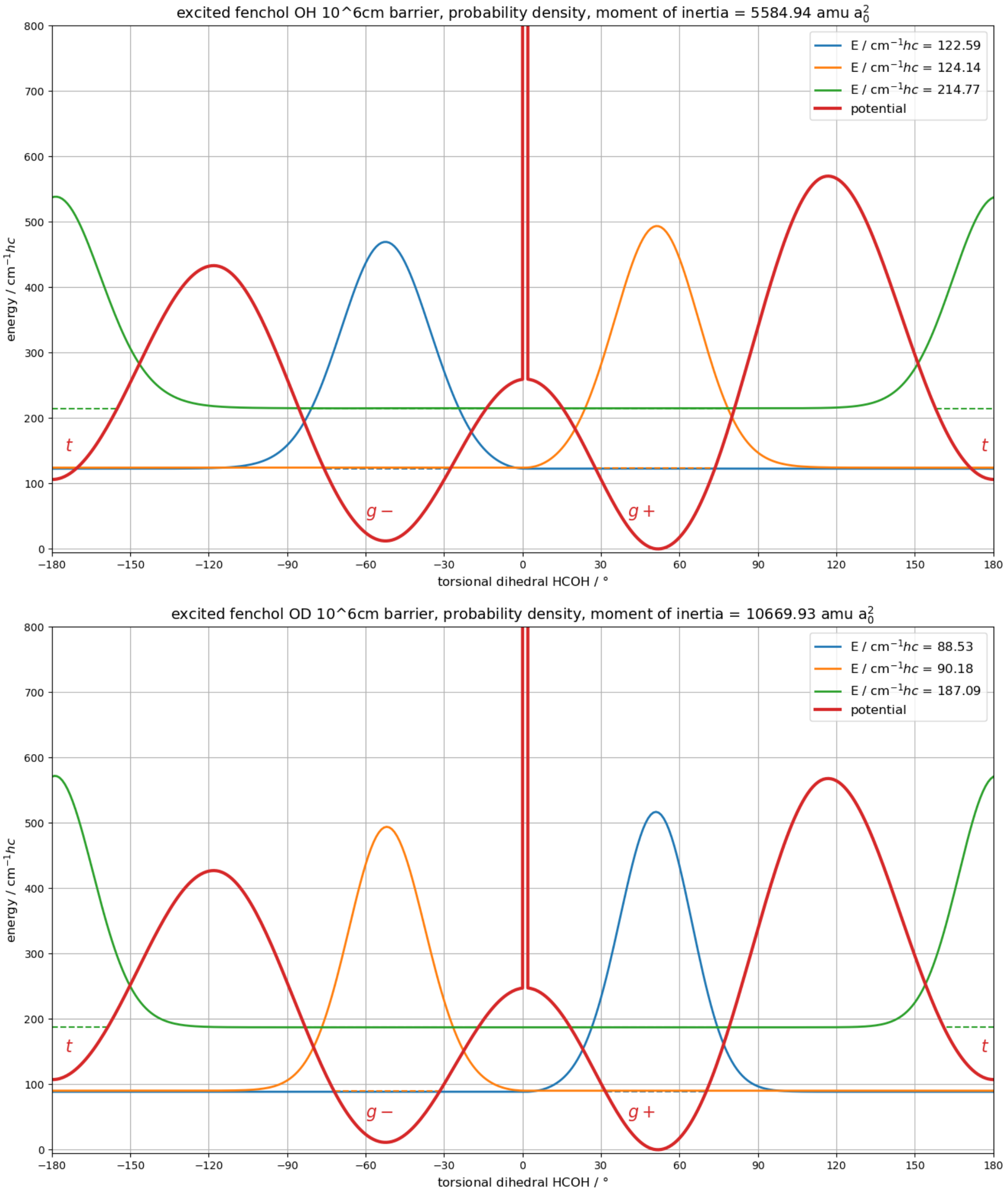

Figure S34: Estimated torsional potential (red trace) of (+)-α-fenchol in the OH (top) or OD (bottom) stretch excited state, based on Figure S30, but with an additional narrow (1°) and towering ($10^6\,cm^{-1}hc$) rectangle barrier at the $g-/g+$ transition state to suppress tunneling.

7.3 Dependence of Torsional Properties on the Asymmetry

7.3.1 (De-)Localization

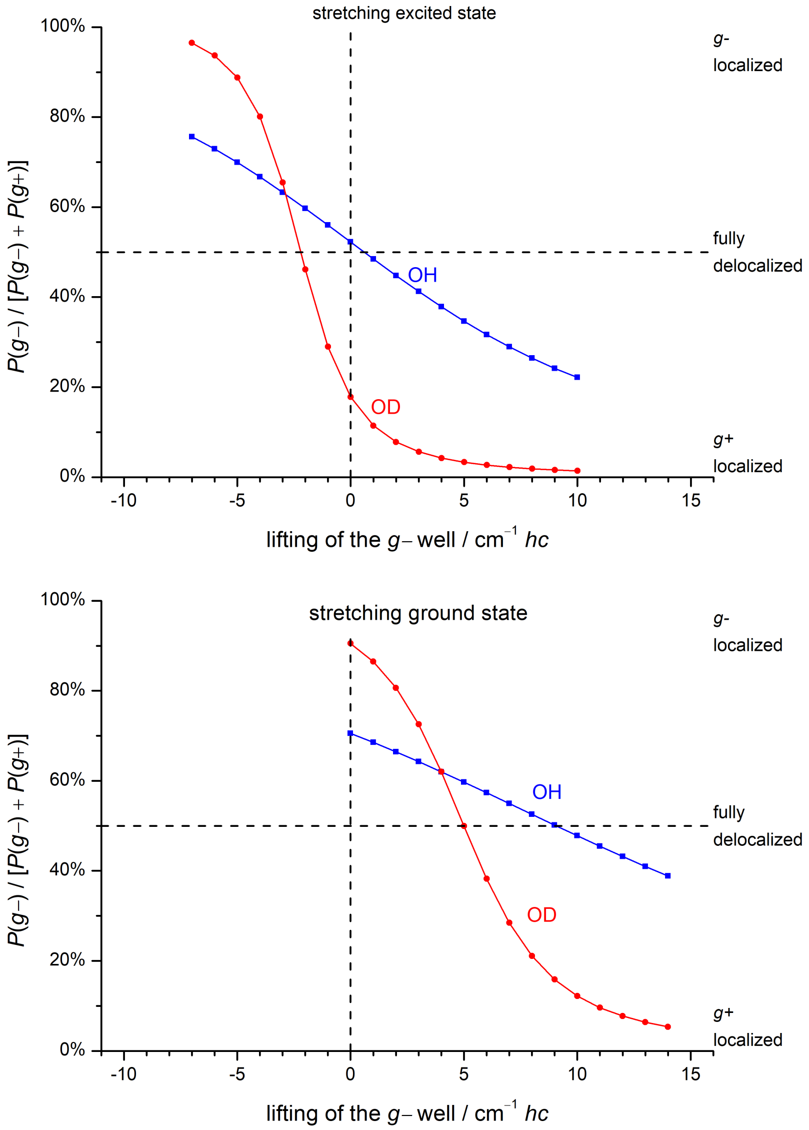

Figure S35: Dependence of the probability density P share in the $g-$ and $g+$ potential wells of the two lowest torsional states of (+)-α-fenchol in the OH/OD ground (bottom) and excited state (top) on the variation of the asymmetry (via lifting of the $g-$ potential minimum before scaling) starting from Figure 10 in the main document and Figure S30.

7.3.2 Franck-Condon Factors

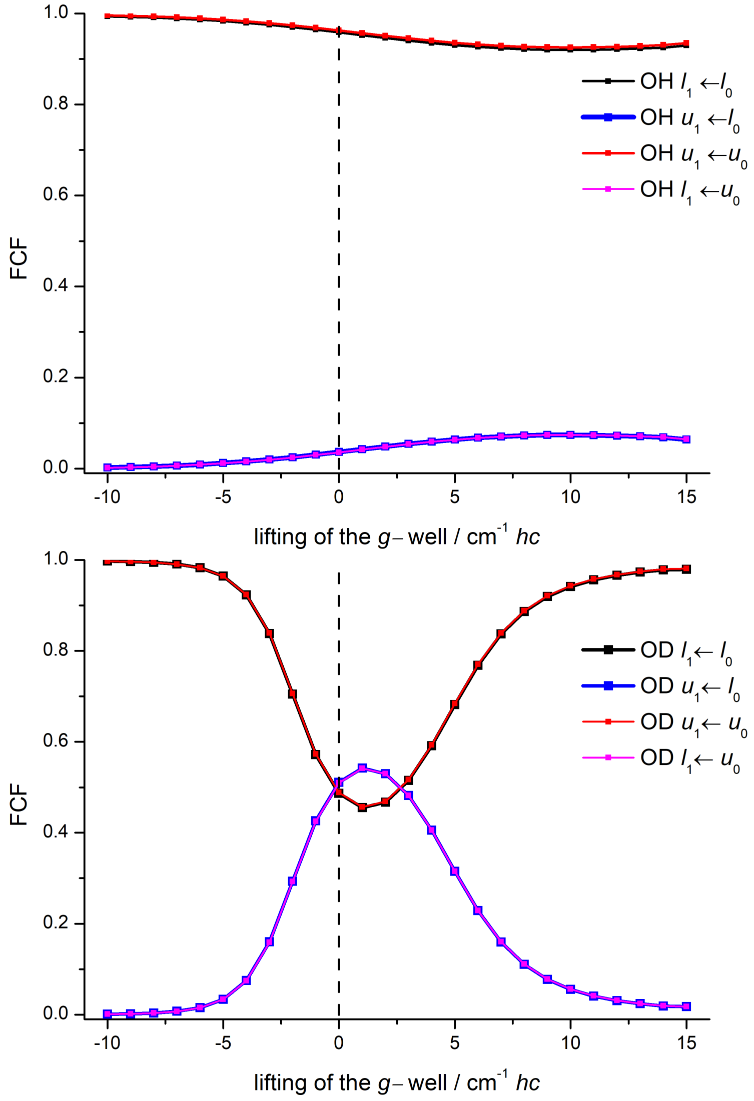

Figure S36: Dependence of the Franck-Condon factors FCFs between the two lowest torsional states of (+)-α-fenchol-OH (top) and -OD (bottom) on the variation of the asymmetry (via concerted lifting of the $g-$ potential minima before scaling) starting from the electronic DLPNO-CCSD(T)/aug-cc-pVQZ//B3LYP-D3(BJ)/may-cc-pVTZ potential (Figure 10 in the main document and Figure S30).

7.3.3 Torsional Splitting

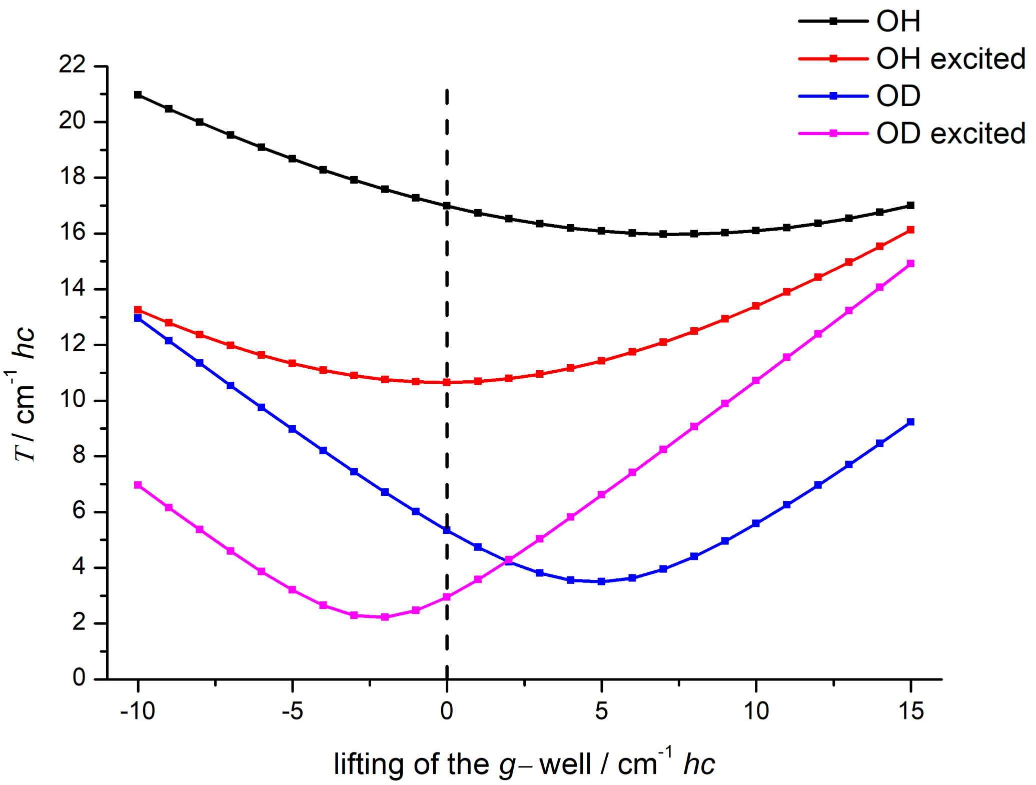

Figure S37: Dependence of the total torsional splitting T between the two lowest states of (+)-α-fenchol (OH and OD as well as stretch ground and excited states) on the variation of the asymmetry (via lifting of the $g-$ potential minimum before scaling) starting from the electronic DLPNO-CCSD(T)/aug-cc-pVQZ//B3LYP-D3(BJ)/may-cc-pVTZ potential (Figure 10 in the main document and Figure S30).

8 Modelling of Methanol in the OH Stretch Ground and Excited State

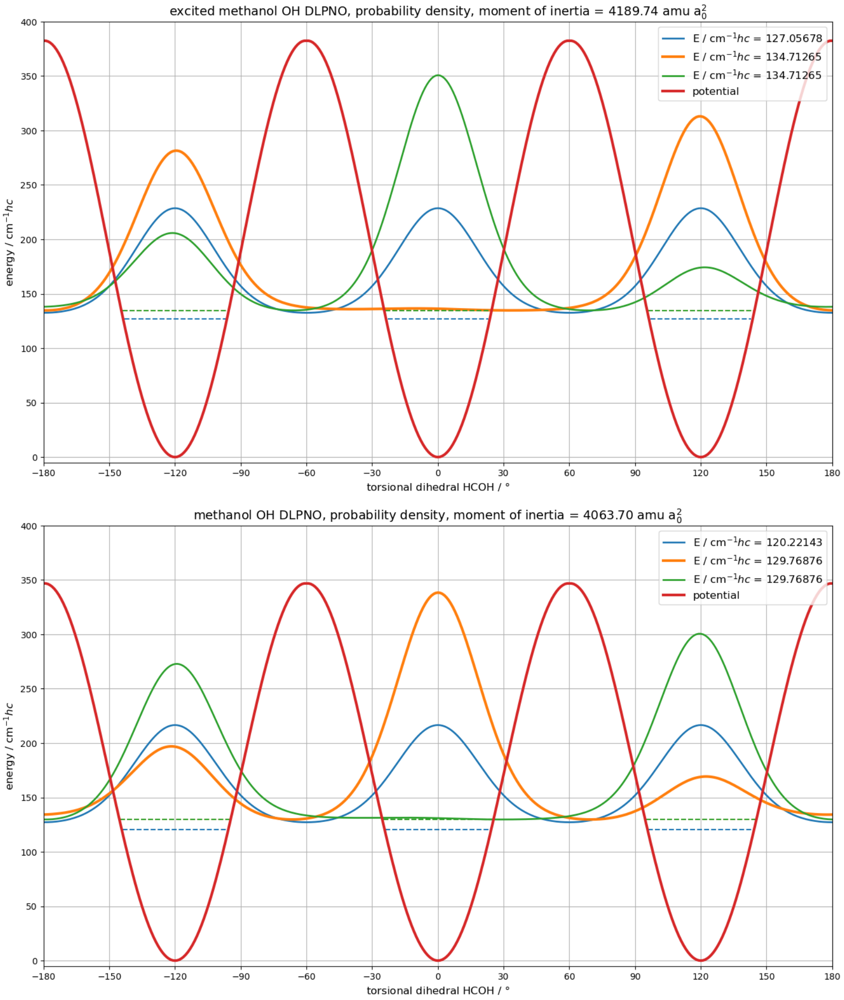

Figure S38: Bottom: Electronic torsional potential (red trace) for the OH stretch ground state of methanol calculated at B3LYP-D3(BJ)/may-cc-pVTZ level and scaled to DLPNO-CCSD(T)/aug-cc-pVQZ single-point corrections for the stationary points. A constant moment of inertia, based on the minimum geometry, was used.
Top: Estimated torsional potential (red trace) for the OH stretch excited state of methanol by also adding the harmonic B3LYP OH stretch wavenumbers at the stationary points before scaling. A constant moment of inertia, based on the minimum geometry, was used.

9 Modelling of Propargyl Alcohol in the OH Stretch Ground and Excited State as well as Franck-Condon Factors

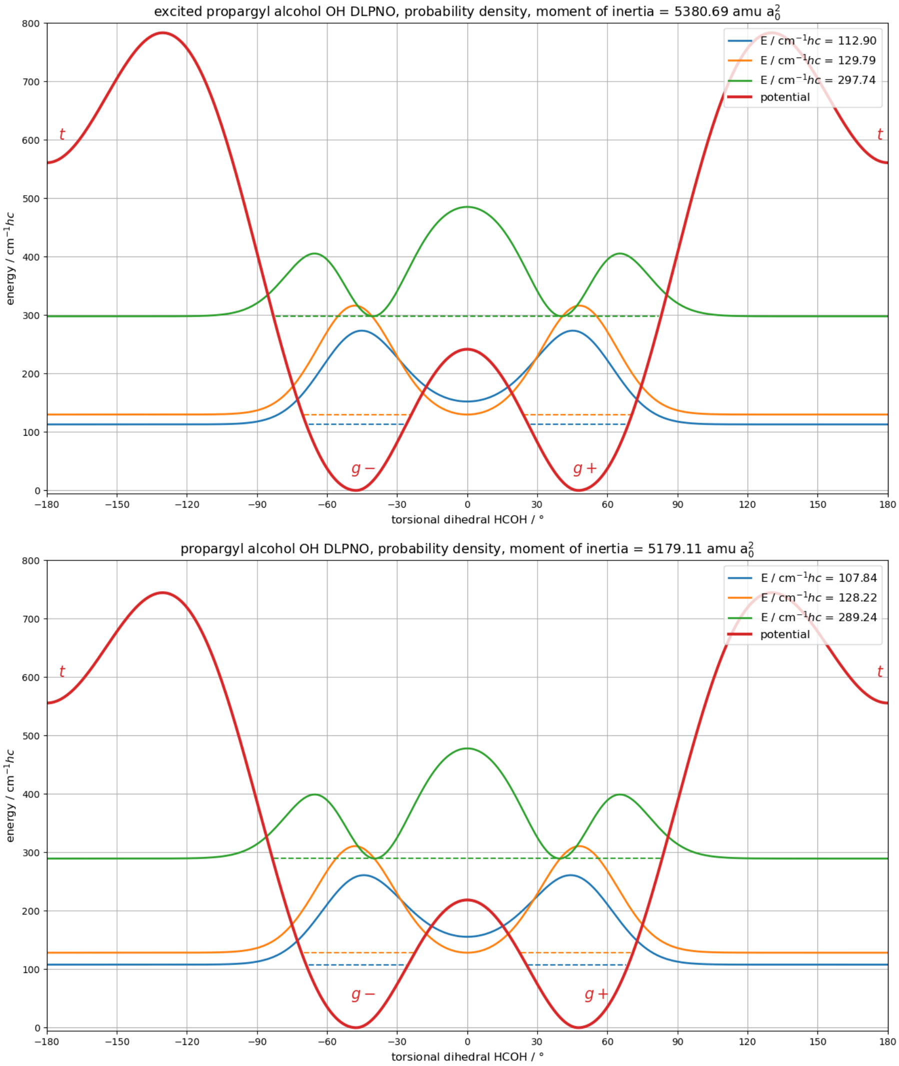

Figure S39: Bottom: Electronic torsional potential (red trace) for the OH stretch ground state of propargyl alcohol calculated at B3LYP-D3(BJ)/may-cc-pVTZ level and scaled to DLPNO-CCSD(T)/aug-cc-pVQZ single-point corrections for the stationary points. A constant moment of inertia, based on the g minimum geometry, was used.
Top: Estimated torsional potential (red trace) for the OH stretch excited state of propargyl alcohol by also adding the harmonic B3LYP OH stretch wavenumber at the stationary points before scaling.

Table S11: Franck-Condon factors $\left\langle \chi^1_{m'} \chi^0_m \right\rangle^2$ for propargyl alcohol based on Figure S39.

	$\chi^1_1 \mathrel{\hat{=}} l_1$	$\chi^1_2 \mathrel{\hat{=}} u_1$	χ^1_3
$\chi^0_1 \mathrel{\hat{=}} l_0$	0.999	$5.03 \cdot 10^{-28}$	$5.14 \cdot 10^{-4}$
$\chi^0_2 \mathrel{\hat{=}} u_0$	$5.79 \cdot 10^{-28}$	1.000	$3.65 \cdot 10^{-27}$
χ^0_3	$5.17 \cdot 10^{-4}$	$3.66 \cdot 10^{-27}$	0.999

10 List of Isomers of Propargyl Alcohol Dimers

Table S12: Isomers of propargyl alcohol dimers in the nomenclature explained in the main document and, where available, notation of Ref. 27 with relative zero-point corrected energy at B3LYP-D3(BJ)/may-cc-pVTZ level. No isomers of the *gt*-hom-$O^g\pi$ or the *gg*-hom-O^tV type could be found as they kept converging into different motifs in all attempts of optimization. Not considered were the πV and VV motifs. There is also the possibility of further OV minima existing with different C_αOO'C'_α dihedrals (describing an internal rotation about the hydrogen bond), which were not explored systematically due to the high energies of all known isomers of this motif and the lack of experimental indications.

notation this work	notation Ref. 27	$E_0/\,\mathrm{kJ\,mol^{-1}}$
gg-hom-$O^g\pi$	Structure 1	0
gg-hom-$O^t\pi$	Structure 5	1.2
gg-het-ππ		1.9
gg-hom-ππ		2.7
gg-het-$O^t\pi$	Structure 5A	2.7
gg-het-$O^g\pi$		2.8
gg-het-O^gV	Structure 3	3.6
gg-het-O^tV	Structure 4	4.1
gg-hom-O^gV	Structure 2	4.3
gt-het-$O^g\pi$	Structure 6	6.9
tg-O^gV		12.6
tg-O^tV		13.9
tt-O^gV	Structure 7	17.6
gt-hom-$O^g\pi$	Structure 6A	not stable
gg-hom-O^tV		not stable

11 Concentration Dependence of the FTIR Jet Spectrum of Propargyl Alcohol

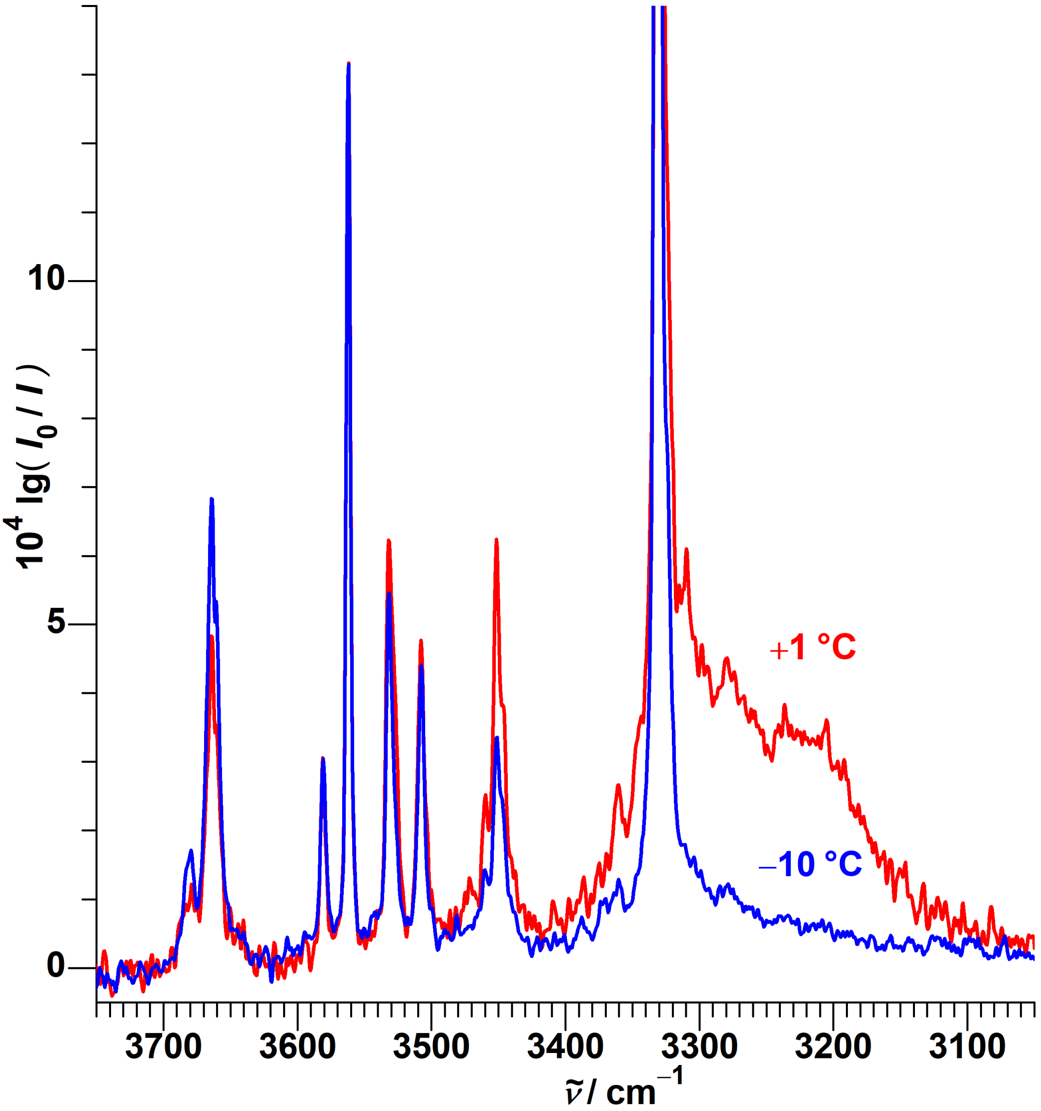

Figure S40: Comparison between FTIR jet spectra in the OH stretching range of propargyl alcohol at two different saturator temperatures with otherwise identical conditions. The spectrum obtained when using −10 °C is scaled by a factor of 2.21 to match the intensity of the band at 3562 cm^{-1} of the spectrum at +1 °C. It can be seen that the four bands between 3600 and 3500 cm^{-1} depend very similarly on the concentration and are therefore assigned to the same cluster size (dimers). Signals at higher wavenumbers scale less steeply with the concentration (assigned to monomers), while signals at lower wavenumber scale more steeply (assigned to larger clusters). The cropped intense signal at 3331 cm^{-1} is the acetylenic CH stretch.

12 Leading Isomers of (+)-α-Fenchol Dimers

Table S13: Properties of leading isomers of (+)-α-fenchol dimers at B3LYP-D3(BJ)/may-cc-pVTZ level obtained from reoptimization of the results from a conformational search at B3LYP-D3(BJ)/def-TZVP level up to 3.1 kJ mol^{-1}. Given are the constituting monomer conformers, the relative zero-point corrected energy as well as the harmonic OH stretching donor and acceptor wavenumbers.

donor	acceptor	E_0/ kJ mol^{-1}	ω_{donor}/ cm^{-1}	ω_{acceptor}/ cm^{-1}
$g-$	t	0	3639	3815
$g+$	t	1.5	3638	3819
$g-$	$g+$	1.8	3636	3805
$g-$	$g-$	1.9	3651	3827
t	t	3.0	3652	3814
$g-$	t	3.2	3612	3814

13 Calculation of Raman Cross Sections

In a first step the Raman activities A_R and depolarisation ratios P from the Gaussian 09 output are converted into the derivatives of the isotropic and anisotropic polarisability α' and γ' according to eqn 1 and 2.[28]

$$\alpha'^2 = \frac{A_\mathrm{R}}{45}\left(1 - \frac{7P}{3P+3}\right) \tag{1}$$

$$\gamma'^2 = \frac{A_\mathrm{R}P}{3P+3} \tag{2}$$

Detection sensitivity for differently polarised light is accounted for by the empirically determined polynomial in eqn 3 (determined by M. Gawrilow, Ref. 29). For $\tilde{\nu}$ the corrected harmonic values are used.

$$\begin{aligned} f(\tilde{\nu}) =& 1.7654 \\ &+ 2.6970 \cdot 10^{-10}\,\mathrm{cm}^3 \cdot (\tilde{\nu} - 2000\,\mathrm{cm}^{-1})^3 \\ &+ 0.4316 \cdot 10^{-13}\,\mathrm{cm}^4 \cdot (\tilde{\nu} - 2000\,\mathrm{cm}^{-1})^4 \\ &- 1.1285 \cdot 10^{-16}\,\mathrm{cm}^5 \cdot (\tilde{\nu} - 2000\,\mathrm{cm}^{-1})^5 \\ &- 0.1278 \cdot 10^{-19}\,\mathrm{cm}^6 \cdot (\tilde{\nu} - 2000\,\mathrm{cm}^{-1})^6 \\ &+ 0.1926 \cdot 10^{-22}\,\mathrm{cm}^7 \cdot (\tilde{\nu} - 2000\,\mathrm{cm}^{-1})^7 \\ &+ 0.1029 \cdot 10^{-26}\,\mathrm{cm}^8 \cdot (\tilde{\nu} - 2000\,\mathrm{cm}^{-1})^8 \\ &- 1.1527 \cdot 10^{-30}\,\mathrm{cm}^9 \cdot (\tilde{\nu} - 2000\,\mathrm{cm}^{-1})^9 \end{aligned} \tag{3}$$

Raman cross sections $\sigma(\tilde{\nu})$ are finally obtained through eqn 4, using the laser wavelength $\lambda_\mathrm{Laser} = 532.27$ nm.

$$\sigma(\tilde{\nu}) = \frac{2\pi^2 h \lambda_\mathrm{Laser}^{-1}}{45c} \cdot \frac{\left(\lambda_\mathrm{Laser}^{-1} - \tilde{\nu}\right)^3}{\tilde{\nu}} \cdot \left(45\alpha'^2 + 4\gamma'^2 + \frac{3\gamma'^2}{f(\tilde{\nu})}\right) \tag{4}$$

14 Used Keywords for Calculations

14.1 Gaussian 09 Rev. E.01

```
B3LYP-D3(BJ)/may-cc-pVTZ geometry optimization:
# B3LYP empiricaldispersion=GD3BJ may-cc-pVTZ int=ultrafine fopt=verytight freq=raman

B3LYP-D3(BJ)/may-cc-pVTZ transition state optimization:
# B3LYP empiricaldispersion=GD3BJ may-cc-pVTZ int=ultrafine opt=(ts,calcfc,noeigen,verytight)

PBE0-D3(BJ)/may-cc-pVTZ geometry optimization:
# PBE1PBE empiricaldispersion=gd3bj may-cc-pvtz int=ultrafine fopt=verytight freq=raman

B2PLYP-D3(BJ)/may-cc-pVTZ geometry optimization:
# B2PLYPD3 may-cc-pvtz int=ultrafine fopt=verytight freq

MP2/6-311++g(d,p) geometry optimization:
# MP2 6-311++g(d,p) scf=tight fopt=verytight freq

B3LYP-D3(BJ)/may-cc-pVTZ torsional scan:
# B3LYP empiricaldispersion=GD3BJ may-cc-pVTZ int=ultrafine opt=modredundant
```

14.2 ORCA version 4.2.1

```
DLPNO-CCSD(T)/aug-cc-pVTZ single-point:
! DLPNO-CCSD(T) TightPNO aug-cc-pVQZ aug-cc-pVQZ/C TightSCF
```

15 Experimental Band Positions and Assignments

Table S14: Experimental band positions and assignments for the spectra of α-fenchol-OH.

$\tilde{\nu}/\mathrm{cm}^{-1}$	assignment
3675	$g\ \ u_1 \leftarrow l_0$
3666	$g\ \ l_1 \leftarrow l_0$
3659	$g\ \ u_1 \leftarrow u_0$
3657	H_2O symmetric stretch
3650	$g\ \ l_1 \leftarrow u_0$
3646	t
3633	dimer acceptor
3499	dimer donor

Table S15: Experimental band positions and assignments for the spectra of α-fenchol-OD.

$\tilde{\nu}/\mathrm{cm}^{-1}$	assignment
2733	overtone/combination
2723	overtone/combination
2723	HDO
2718	MeOD
2713	unassigned
2707	$g\ \ u_1 \leftarrow l_0$
2704	$g\ \ l_1 \leftarrow l_0$
2700	$g\ \ u_1 \leftarrow u_0$
2697	$g\ \ l_1 \leftarrow u_0$
2689	t
2680	dimer acceptor
2671	D_2O symmetric stretch
2585	dimer donor

Table S16: Experimental band positions and assignments for the spectra of propargyl alcohol.

$\tilde{\nu}/\mathrm{cm}^{-1}$	assignment
3680	$g\ \ u_1 \leftarrow l_0$
3662	$g\ \ l_1 \leftarrow l_0$
3658	$g\ \ u_1 \leftarrow u_0$
3640	$g\ \ l_1 \leftarrow u_0$
3581	gg-hom-$O^t\pi$ $OH\cdots\pi$
3562	gg-hom-$O^g\pi$ out-of-phase
3532	gg-hom-$O^g\pi$ in-phase
3507	gg-hom-$O^t\pi$ $OH\cdots O$
3452	larger clusters (trimers?)
3331	acetylenic CH stretch
3400–3100	larger clusters

References

[1] R. Medel and M. A. Suhm, *Phys. Chem. Chem. Phys.*, 2021, **23**, 5629–5643.

[2] M. Heger, *Diagonal and Off-Diagonal Anharmonicity in Hydrogen-Bonded Systems*, Dissertation, Georg-August-Universität-Göttingen, 2016.

[3] P. Zielke, *Ramanstreuung am Überschallstrahl: Wasserstoffbrückendynamik aus neuer Perspektive*, Dissertation, Georg-August-Universität Göttingen, 2007.

[4] T. N. Wassermann and M. A. Suhm, *J. Phys. Chem. A*, 2010, **114**, 8223–8233.

[5] T. N. Wassermann, M. A. Suhm, P. Roubin and S. Coussan, *J. Mol. Struct.*, 2012, **1025**, 20–32.

[6] G. Dieke and H. Crosswhite, *J. Quant. Spectrosc. Radiat. Transf.*, 1962, **2**, 97–199.

[7] J. A. Coxon, *J. Mol. Spectrosc.*, 1975, **58**, 1–28.

[8] G. Moruzzi, L. Xu, R. Lees, B. Winnewisser and M. Winnewisser, *J. Mol. Spectrosc.*, 1994, **167**, 156–175.

[9] I. Mukhopadhyay, Y. Duan and K. Takagi, *Spectrochim. Acta. A. Mol. Biomol. Spectrosc.*, 1998, **54**, 1325–1335.

[10] E. L. Sibert and J. Castillo-Chará, *J. Chem. Phys.*, 2005, **122**, 194306.

[11] L.-H. Xu, H. Müller, F. van der Tak and S. Thorwirth, *J. Mol. Spectrosc.*, 2004, **228**, 220–229.

[12] R. K. Kakar and C. R. Quade, *J. Chem. Phys.*, 1980, **72**, 4300–4307.

[13] J. Pearson and B. Drouin, *J. Mol. Spectrosc.*, 2005, **234**, 149–156.

[14] L. Margulès, B. A. McGuire, M. L. Senent, R. A. Motiyenko, A. Remijan and J. C. Guillemin, *Astron. Astrophys.*, 2017, **601**, A50.

[15] G. Cazzoli, D. G. Lister and A. M. Mirri, *J. Chem. Soc. Faraday Trans. 2*, 1973, **69**, 569.

[16] R. A. Motiyenko, L. Margulès, M. L. Senent and J.-C. Guillemin, *J. Phys. Chem. A*, 2018, **122**, 3163–3169.

[17] S. Melandri, P. G. Favero and W. Caminati, *Chem. Phys. Lett.*, 1994, **223**, 541–545.

[18] R. A. Motiyenko, L. Margulès, D. Despois and J.-C. Guillemin, *Phys. Chem. Chem. Phys.*, 2018, **20**, 5509–5516.

[19] R. Suenram, F. Lovas and H. Pickett, *J. Mol. Spectrosc.*, 1986, **119**, 446–455.

[20] L.-H. Xu, G. T. Fraser, F. J. Lovas, R. D. Suenram, C. W. Gillies, H. E. Warner and J. Z. Gillies, *J. Chem. Phys.*, 1995, **103**, 9541–9548.

[21] E. Hirota and Y. Kawashima, *J. Mol. Spectrosc.*, 2001, **207**, 243–253.

[22] M. Juanes, W. Li, L. Spada, L. Evangelisti, A. Lesarri and W. Caminati, *Phys. Chem. Chem. Phys.*, 2019, **21**, 3676–3682.

[23] J. N. Macdonald, D. Norbury and J. Sheridan, *J. Chem. Soc. Faraday Trans. 2*, 1978, **74**, 1365.

[24] E. Cohen, B. Drouin, E. Valenzuela, R. Woods, W. Caminati, A. Maris and S. Melandri, *J. Mol. Spectrosc.*, 2010, **260**, 77–83.

[25] W. Li, L. Spada, L. Evangelisti and W. Caminati, *J. Phys. Chem. A*, 2016, **120**, 4338–4342.

[26] A. Leonov, K.-M. Marstokk, A. de Meijere and H. Møllendal, *J. Phys. Chem. A*, 2000, **104**, 4421–4428.

[27] J. Saini and K. S. Viswanathan, *J. Phys. Chem. A*, 2017, **121**, 1448–1459.

[28] N. O. B. Lüttschwager, *Raman Spectroscopy of Conformational Rearrangements at Low Temperatures Folding and Stretching of Alkanes in Supersonic Jets*, Springer International Publishing, Cham, 2014.

[29] M. Gawrilow and M. A. Suhm, *Phys. Chem. Chem. Phys.*, 2020, **22**, 15303–15311.
